# Comparative proteomics of biofilm development in *Pseudoalteromonas tunicata* discovers a distinct family of Ca^2+^-dependent adhesins

**DOI:** 10.1128/mbio.01069-25

**Published:** 2025-05-21

**Authors:** Sura Ali, Alexander Stavropoulos, Benjamin Jenkins, Sadie Graves, Atiyeh Ahmadi, Vania Marzbanrad, Geoffrey Che, Jiujun Cheng, Huagang Tan, Xin Wei, Suhelen Egan, Brian Ingalls, Josh D. Neufeld, Ulrich Eckhard, Trevor C. Charles, Andrew C. Doxey

**Affiliations:** 1 Department of Biology and Waterloo Centre for Microbial Research, University of Waterloo 98689, Waterloo, Ontario, Canada; 2 The University of New South Wales Sydney 7800, Sydney, New South Wales, Australia; 3 Department of Applied Mathematics, University of Waterloo 153522, Waterloo, Ontario, Canada; 4 Synthetic Structural Biology Group, Department of Molecular and Structural Biology, Molecular Biology Institute of Barcelona (IBMB-CSIC) 98693 , Barcelona, Catalonia, Spain; Indiana University Bloomington, Bloomington, Indiana, USA

**Keywords:** biofilms, proteomics, adhesins, bioinformatics, marine microbiology

## Abstract

The marine bacterium, *Pseudoalteromonas tunicata*, is a useful model for studying biofilm development due to its ability to colonize and form biofilms on a variety of marine and eukaryotic host-associated surfaces. However, the pathways responsible for *P. tunicata* biofilm formation are not fully understood, in part due to a lack of functional information for a large proportion of its proteome. We used comparative shotgun proteomics to explore *P. tunicata* biofilm development from the planktonic phase throughout early, middle, and late biofilm stages. A total of 248 biofilm-associated proteins were identified, including many hypothetical proteins, as well as previously known *P. tunicata* biofilm-related proteins, such as the autocidal enzyme AlpP, violacein proteins, S-layer protein SLR4, and various pili proteins. We further investigated the top identified biofilm-associated protein, a previously uncharacterized 1,600-amino acid protein (EAR30327), which we designate as “BapP.” Based on AlphaFold modeling and genomic context analysis, we predicted BapP as a distinct Ca^2+^-dependent biofilm adhesin. Consistent with this prediction, a Δ*bapP* knockout mutant was defective in forming both pellicle- and surface-associated biofilms and rescued by re-insertion of *bapP* into the genome. Similar to the mechanisms of RTX Bap-like adhesins, BapP-mediated biofilm formation was influenced by Ca^2+^ levels, and BapP is potentially exported by a Type 1 secretion system. Ultimately, our work not only provides a useful proteomic data set for studying biofilm development in an ecologically relevant organism but also adds to our knowledge of bacterial adhesin diversity, emphasizing Bap-like proteins as widespread determinants of biofilm formation in bacteria.

**IMPORTANCE:**

Understanding how bacteria form biofilms is essential because biofilms play a crucial role in bacterial survival and interaction with their environments. The marine bacterium *Pseudoalteromonas tunicata* is a valuable model for studying biofilm formation, as it colonizes diverse marine surfaces and host organisms. By identifying proteins involved in biofilm development, our study sheds light on the specific proteins that help *P. tunicata* transition from a free-swimming state to a stable biofilm. This work highlights the role of a large, calcium-dependent protein, BapP, which we found to be essential for biofilm stability and structure. This protein and hundreds of others identified provide new insights into bacterial adhesion mechanisms, expanding our understanding of biofilm formation in marine environments and potentially informing broader studies on biofilm-related processes in other bacteria.

## INTRODUCTION

Understanding the molecular processes that control biofilm development by environmental and host-associated microorganisms is a fundamental area of microbiology, with important industrial and ecological applications. Members of the *Pseudoalteromonas* genus (class *Gammaproteobacteria*) are commonly found in marine environments in association with biological surfaces and diverse eukaryotic hosts and play important roles in the ecology of marine ecosystems (1–3). One of the best studied species within this group is *Pseudoalteromonas tunicata,* a heterotrophic, gram-negative bacterium first isolated from the tunicate, *Ciona intestinalis* (4). *P. tunicata* is also known to colonize algal host surfaces, sea-water biofilm communities, and likely other yet-to-be-identified living surfaces and host organisms in the marine environment (5, 6). Among *P. tunicata*’s characteristics is its ability to colonize and outcompete other species in natural biofilms (6, 7), which is in part due to its broad repertoire of antimicrobial capabilities (3–5, 8). Characterizing the molecular basis of *P. tunicata*’s biofilm formation, colonization, and antifouling activity is important not only in the context of understanding marine biofilm ecology (6–9), but it could also reveal new biotechnological strategies for preventing harmful biofilm formation (e.g., in industrial settings or infections) (10, 11).

Several previous studies have investigated biofilm development in *P. tunicata* (6–8), which shares several general mechanisms with that of other biofilm-forming bacteria. The first step is the initial attachment/adhesion to surfaces (8). Typically, these initial interactions involve weak and reversible binding to a surface substrate using adhesive structures, such as flagella and pili (12). The genome of *P. tunicata* encodes a variety of flagellar genes, including a proteolytically active variant of flagellin that might be involved in flagella-mediated interactions with surfaces or host cells (13, 14). In addition, the *P. tunicata* genome encodes a diversity of surface adhesion-related proteins capable of binding diverse substrates in the marine environment. These include type IV pili, curli, MSHA-like pili, capsular polysaccharide, chitin and cellulose-binding proteins, as well as specialized proteins for binding to extracellular matrix (ECM) components abundant in eukaryotic host surfaces (5, 15, 16). Among these is LipL32, which facilitates adhesion of *P. tunicata* to the ECM of its *C. intestinalis* host (17).

The transition of *P. tunicata* to a surface-associated lifestyle involves dynamic changes in its transcriptome and proteome (15). These changes include the production of anti-biofouling agents (e.g., antilarval, antibacterial, antialgal, and antifungal molecules) (3, 18), as well as the formation of the extracellular biofilm matrix that provides mechanical and protective scaffold for the biofilm. The ToxR-like transcriptional regulator WmpR is a key regulatory protein that controls stationary-phase expression of antifouling inhibitors in *P. tunicata* (19). WmpR also controls the production of other bioactive compounds and pigments (19) and other proteins associated with adaptation to a biofilm lifestyle, such as iron acquisition genes and type IV pili (8, 18). One of the key proteins produced by *P. tunicata* is the autocidal enzyme, AlpP, that causes cell lysis within biofilms (8). AlpP is a lysine oxidase that produces hydrogen peroxide (20) and exhibits antibacterial activity against other gram-negative and -positive bacteria (21). The controlled AlpP-mediated autolysis of subpopulations of cells within the center of biofilm microcolonies is thought to promote the detachment of dense clusters of cells, regulate biofilm spatial architecture, and facilitate dispersal (8). Although the extracellular biofilm matrix is relatively uncharacterized in *P. tunicata*, a recent study identified and characterized a novel protein, SLR4, as an abundant matrix component (22). SLR4 not only forms the protective S-layer around cells in a planktonic state but also around extracellular components of the biofilm matrix, including outer membrane vesicles and filaments (22).

Despite previous knowledge of biofilm development in *P. tunicata*, there exist hundreds of proteins of unknown function encoded within the *P. tunicata* genome (5), many of which may play important biofilm-related functions. Here, to further elucidate the molecular mechanisms and identify key proteins responsible for biofilm development in *P. tunicata*, we have performed a time-course shotgun proteomic analysis of *P. tunicata* cells from planktonic to early and late biofilm states. Our analysis reveals hundreds of candidate biofilm-associated proteins, including many hypothetical proteins of unknown function. We then investigated the top-scoring biofilm-associated protein, EAR30327 (which we designated as “BapP”), and characterized its function using reverse genetic methods. Our work establishes BapP as a novel Ca^2+^-dependent adhesin required for biofilm formation in *P. tunicata*, thus contributing to our understanding of marine biofilm development and the diversity of Bap-like biofilm adhesins in bacteria.

## RESULTS AND DISCUSSION

### Proteomic analysis of biofilm development in *P. tunicata*

**Fig 1:**
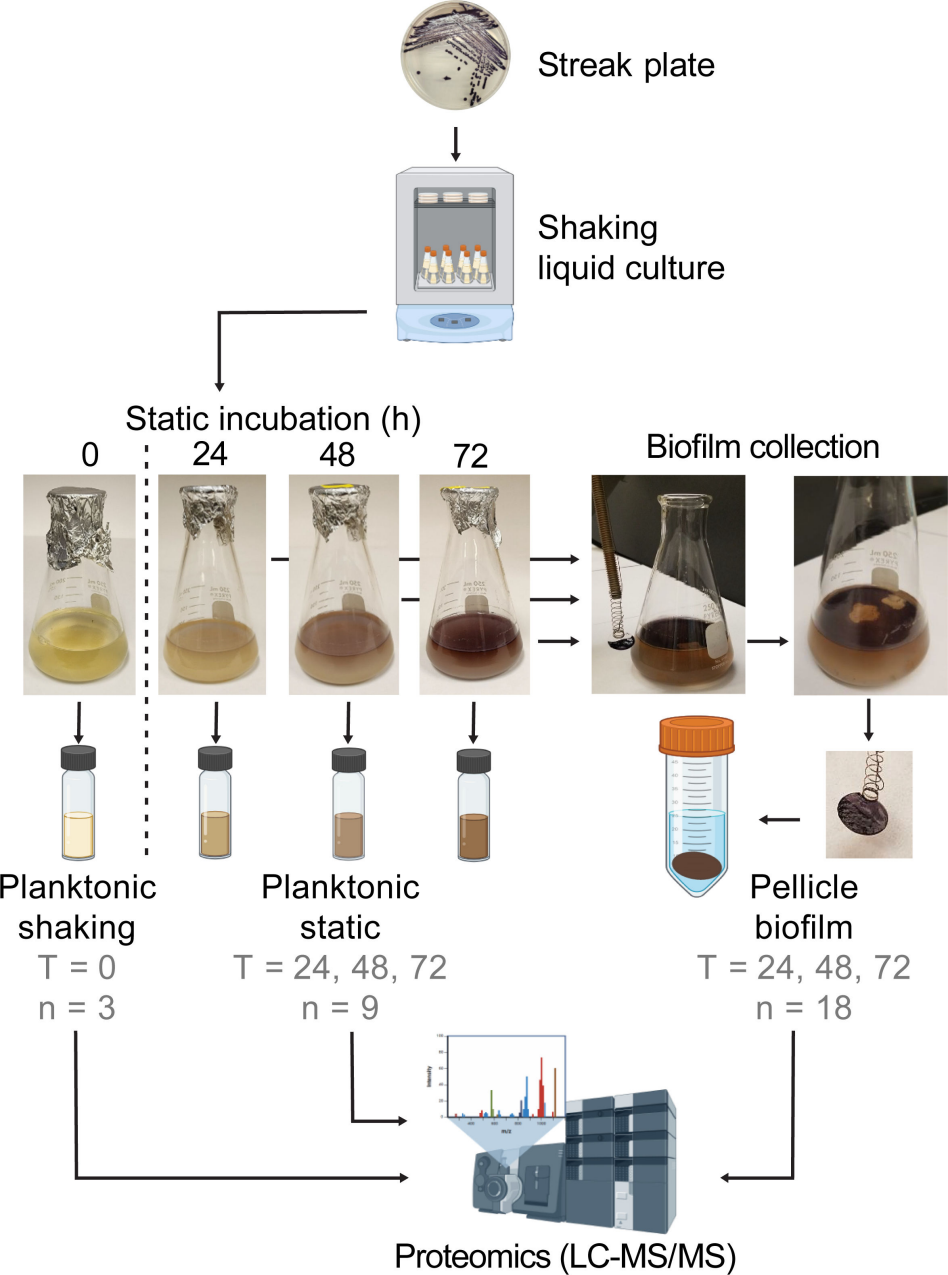
Overview of protocol for sampling of *P. tunicata* pellicle biofilms and planktonic cultures. First, *P. tunicata* was cultured on marine agar streak plates. Overnight cultures were subcultured and incubated in shaking conditions at 24°C for 8 h to generate “planktonic shaking” samples. These samples were then used to make liquid cultures in marine broth. In static (non-shaking) conditions, pellicle biofilms began to develop after approximately 24 h and increased in biomass over time at the air-liquid interface. “Pellicle biofilm” samples were collected at 24, 48, and 72 h. A device was used to extract circular segments from the pellicle biofilm, which was resuspended in Tris-HCl, pH 8.3, and used for liquid chromatography tandem mass spectrometry (LC-MS/MS) proteomic analysis. For comparison, “planktonic static” samples of liquid media below the biofilm surface at each time point were also taken for proteomic analysis.

**Fig 2:**
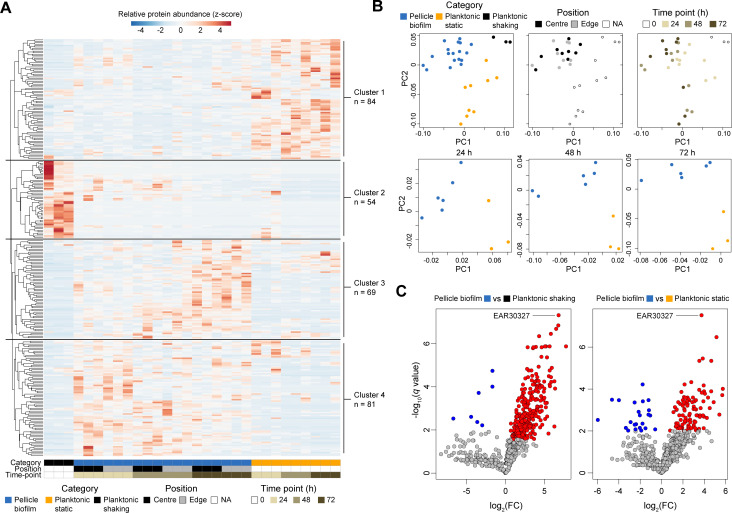
Comparative proteomic analysis of *Pseudoalteromonas tunicata* throughout biofilm development. (**A**) Proteomic abundance heatmap of 288 proteins detected by liquid chromatography tandem mass spectrometry analysis. Per-protein abundances (# spectral hits) were row-normalized to Z-scores depicting relative abundance across samples. Proteins clustered into four groups based on their relative abundance profiles across samples. Cluster 1 proteins are planktonic static-enriched; cluster 2 proteins are planktonic shaking-enriched; and cluster 3 and 4 proteins are biofilm-enriched, with cluster 3 associated with mid-to-late stage biofilms and cluster 3 associated with early stage biofilms. (**B**) Principal component analysis (PCA) plot of samples based on their proteomic profiles. Similar to hierarchical clustering in panel **A**, the PCA plot reveals the grouping of samples based on sample type. (**C**) Volcano plots depicting differentially abundant proteins in biofilm samples (*N* = 18) versus planktonic shaking (*N* = 3) samples, as well as planktonic static samples (*N* = 9). Proteins with significantly increased abundance in biofilms are located in the top right quadrant of the plots, with the top-scoring proteins (*q* < 0.01) shown in red. Proteins with significantly increased abundance in shaking planktonic conditions are located in the top left quadrant (colored blue). Non-significant proteins are shown in gray.

To investigate the proteomic determinants involved in *P. tunicata* biofilm development, we conducted comparative LC-MS/MS shotgun proteomics of *P. tunicata* D2 in its transition from a planktonic to a biofilm state (Fig. 1). Liquid cultures were grown for 8 h under shaking conditions to produce “planktonic shaking” samples, and then incubated further under static conditions for 24, 48, and 72 h to produce both “pellicle biofilm” samples, as well as “planktonic static” samples, collected beneath the pellicle (see Materials and Methods). Pellicle biofilms developed as early as 24 h in static conditions and by 72 h became denser and darker in color due to the production of *P. tunicata*’s natural pigments (Fig. 1) (23). Across all samples, we identified a total of 942 *P*. *tunicata* proteins with a coverage of at least one high-confidence peptide assignment (Table S1). A subset of 288 proteins excluding low-abundance proteins was used to generate a heatmap of relative abundance across all samples (Fig. 2A). Proteins clustered into four groups based on their relative abundance profiles: cluster 1 (*n* = 84) proteins were enriched in planktonic static samples; cluster 2 (*n* = 54) proteins were enriched in planktonic shaking samples; and cluster 3 (*n* = 69) and 4 (*n* = 81) proteins were biofilm-associated, with cluster 4 proteins showing increased abundance at early biofilm stages (24 h) and cluster 3 proteins showing increased abundance at middle to late biofilm stages (i.e., 48–72 h). Planktonic shaking, planktonic static, and pellicle biofilm samples, therefore, display unique proteomic profiles. To test this further, we performed principal component analysis (PCA) of all samples based on their proteomic profiles (Fig. 2B). As shown by the PCA, the samples separated distinctly according to their category (planktonic shaking, planktonic static, biofilm; Fig. 2B). Biofilm samples also showed some additional separation by time point consistent with the heatmap but not based on the region of biofilm collected (center versus edge) (Fig. 2B). Although previous studies have demonstrated spatial expression differences in biofilms (24), our inability to detect such differences was likely due to a lack of precision in collecting cells at the biofilm-substrate interface.

While the heatmap provides a visual overview of protein abundance profiles, differential abundance analysis was required to detect statistically significant differences. We, therefore, compared normalized protein abundance across biofilm and non-biofilm samples to detect putative biofilm-associated proteins (Fig. 2C). Out of the 942 detected proteins, 232 had significant normalized abundance increases (log_2_FC > 0.5 and *q* < 0.01, two-tailed *t*-test, BH adjustment of *P* values) in biofilm versus planktonic shaking samples (Fig. 2C; Table S2). Using the same criteria, 100 proteins were significantly more abundant in biofilm samples than in planktonic static cells collected from the same samples (beneath the pellicle biofilms), which may include a mixture of planktonic and detached/dispersed cells from the biofilm (Table S3). A total of 248 unique biofilm-associated proteins were identified across both lists (Table S2 and S3), 84 of which were shared and thus indicate proteins with the strongest association with the pellicle (Table S4). Only 30 proteins were detected with increased abundance in planktonic samples (Fig. 2C; Table S2 and S3), which may be due to lower overall proteomic coverage and fewer biological replicates (*n* = 3 planktonic shaking and *n* = 9 planktonic static) of these samples.

**Fig 3:**
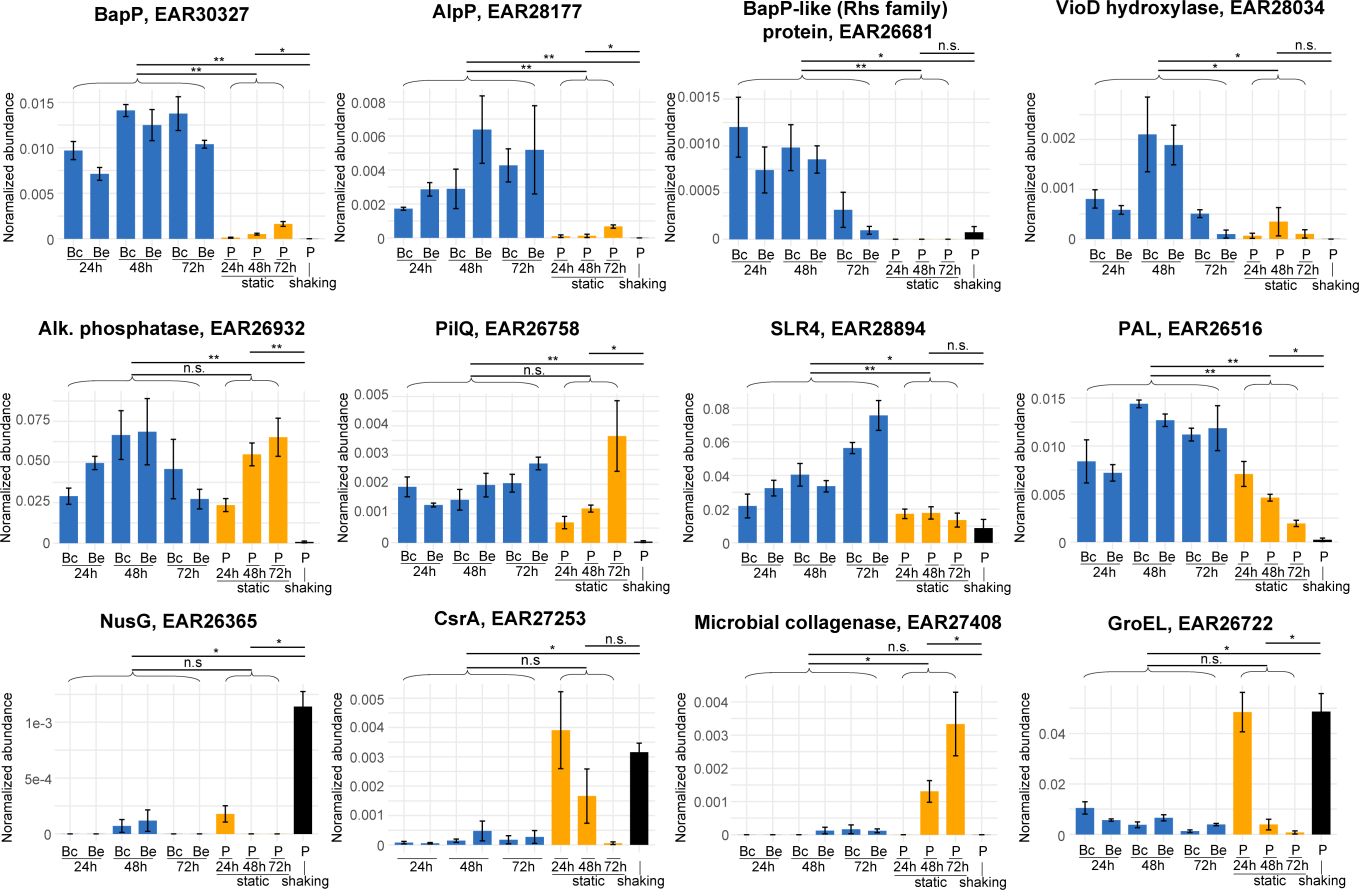
Relative abundance of selected proteins across planktonic shaking, planktonic static, and pellicle biofilm samples based on liquid chromatography tandem mass spectrometry proteomic analysis. Protein abundance was calculated as a percentage of total peptide spectral matches to the *P. tunicata* proteome. Bc—biofilm (center region); Be—biofilm (edge region). Significance levels of pairwise *T*-test comparisons are indicated above the bar plots with ** indicating *P* < 0.0005 and * indicating *P* < 0.05. There were *n* = 3 replicates per condition, and error bars represent the mean ± standard error.

Planktonic shaking-associated proteins included proteins involved in transcription (e.g., NusG) and translation (ribosomal proteins, CsrA). These proteins reflect core physiological processes of intracellular proteins and indicate differences in the metabolic state of cells. CsrA, for example, is a major regulator of biofilm formation by controlling the expression of biofilm genes and c-di-GMP metabolism (25). LC-MS/MS abundance profiles of example planktonic-associated proteins (NusG and CsrA) are shown in Fig. 3. We also detected 20 proteins with significant abundance increases in planktonic static samples (Table S5). These proteins also reflect the activities of non-adherent planktonic cells and, potentially, dispersal cell populations from the biofilm. Several proteins were detected at high levels in 24 h planktonic static samples, as well as in planktonic shaking cells (e.g., GroEL chaperone, Fig. 3).

### Analysis of biofilm-associated proteins

We then explored the top identified biofilm-associated proteins from Table S2 and S3 based on *P* value (see Table 1 for top 10), which include known biofilm-related proteins, as well as proteins with yet-to-be-identified roles. The top-ranked protein overall in both Table S2 and S3 was National Center for Biotechnology Information (NCBI)/GenBank accession #EAR30327 (Fig. 2C; Table 1), a hypothetical protein that we targeted for subsequent experimental characterization. EAR30327 was detected at high relative abundance at all biofilm time points and reduced abundance in the planktonic samples (Fig. 3). The #2 ranked biofilm-associated protein in Table S2 and #39 in Table S3 was peptidoglycan-associated lipoprotein (PAL), a protein known for its role in stabilizing the outer membrane by interacting with the Tol-Pal system (26). The PAL protein showed a similar abundance profile to EAR30327 but in planktonic static samples was found at higher abundance at earlier (24 h) time points (Fig. 3). Although the Tol-Pal system has been shown to be up-regulated during biofilm development in other organisms (26, 27), the considerable abundance of PAL in *P. tunicata* biofilms suggests an important yet-to-be-identified role. One possibility is a role related to the formation or structure of outer membrane vesicles, which are abundant in the biofilm matrix (22). The type IV pilus secretin PilQ (ranked #5 in Table S2) was also significantly increased in biofilm samples and planktonic static samples (Fig. 3). Type IV pili contribute to surface binding and sensing during biofilm formation and may also be involved in the uptake of extracellular compounds and DNA (28). The chaperone DnaJ (ranked #3 in Table S3 and #8 in Table S2) was increased in biofilm development, consistent with a previous study demonstrating that *E. coli* Δ*dnaJ* mutants are deficient in biofilm formation (29). Ranked #9 in Table S2 and #49 in Table S3 was a 2′ 3′-cyclic nucleotide 2′-phosphodiesterase/3′-nucleotidase, and its increased abundance in biofilm may reflect a putative role of nucleotidases in hydrolyzing extracellular DNA for nutrition (30). Ranked #6 in Table S2 was a predicted peptidyl-prolyl *cis*-*trans* isomerase. The related enzyme Ppi-B has been shown to contribute to biofilm formation in *Mycobacterium tuberculosis* (31).

**TABLE 1:** Top 10 proteins with increased abundance in pellicle biofilms compared to planktonic samples^*a*^

Rank	Biofilm vs planktonic shaking		Biofilm vs planktonic static		Accession	Description
	*q*-Value	Log2FC	*q*-Value	log2FC		
1	4.8 × 10^−8^	6.8	3.0 × 10^−8^	3.7	EAR30327	Ig-like domain-containing protein, “BapP” (this study)
2	1.4 × 10^−7^	6.8	7.3 × 10^−4^	1.3	EAR26516	Peptidoglycan-associated protein
3	2.1 × 10^−7^	6.5	3.4 × 10^−7^	5.1	EAR30706	RimK family alpha-L-glutamate ligase
4	3.5 × 10^−7^	5.8	6.0 × 10^−5^	2.2	EAR27711	S46 family peptidase
5	1.3 × 10^−6^	5.1	9.5 × 10^−3^	0.8	EAR26410	FKBP-type peptidyl-prolyl cis-trans isomerase
6	1.3 × 10^−6^	5.7	3.5 × 10^−6^	3.9	EAR30476	Chaperone protein DnaJ
7	1.3 × 10^−6^	7.8	1.3 × 10^−3^	1.1	EAR30609	2′,3′-Cyclic nucleotide 2′-phosphodiesterase/3′-nucleotidase bifunctional periplasmic protein
8	1.5 × 10^−6^	4.7	4.5 × 10^−6^	4.7	EAR28309	Nitroreductase family protein
9	1.6 × 10^−6^	3.5	4.5 × 10^−6^	3.5	EAR28471	Pyrimidine/purine nucleoside phosphorylase
10	4.3 × 10^−6^	5.3	9.8 × 10^−4^	1.4	EAR26625	Uncharacterized protein

^*a*^ The proteins were first ranked by *q*-values based on comparison of LC-MS/MS protein abundances in biofilms versus planktonic shaking samples. These proteins were then filtered to those with significant (*q* < 0.01) abundance increases in biofilm samples compared to planktonic static samples. Descriptions are based on annotations of numerous identical proteins collected from National Center for Biotechnology Information’s “Identical Protein Groups” resource.

Some additional notable biofilm-associated proteins were two alkaline phosphatases (EAR26932, EAR26933) that were particularly abundant in biofilm samples (Tables S1 and S2). Alkaline phosphatases have been shown to be critical for regulating biofilm formation in other organisms (e.g., *P. aeruginosa*) under phosphate depletion stress (32). Ranked #28 in Table S2 was the flagellar hook protein, FlgE, which has been shown to be essential for biofilm formation (33). Other flagellar proteins, including two flagellins (EAR29565, EAR29563), were also enriched in biofilm samples, as well as being detected in the surrounding planktonic static samples. Detection of flagellar proteins was expected given the role of motility in initial biofilm formation, intra-biofilm motility, biofilm dispersal, as well as the potential role of flagellins as structural components of the extracellular and biofilm matrix (34).

Among the list of 248 biofilm-associated proteins, we also detected several proteins previously shown to be important for biofilm development in *P. tunicata*. For example, the MSHA pilin protein MshA had increased biofilm abundance, and the violacein pathway proteins (VioA, C, D, and E) were also significantly increased (Tables S2 and S3). The tryptophan hydroxylase (VioD) protein showed a unique temporal pattern of expression because it was at higher abundance at the middle biofilm (48 h) time point (Fig. 3). The production of such pigments in *P. tunicata* is correlated with antifouling activity (23) and has been observed in mature biofilms (7). The autolytic protein, AlpP, was also detected at significantly higher abundance in biofilms, consistent with previous literature (7, 8). AlpP relative abundance increased over time in biofilms, reaching a maximum level in 72 h biofilms (Fig. 3). This finding is consistent with its role in programmed cell lysis to enable cell redistribution and dispersal (8), which likely increases during later stages of biofilm development. The S-layer protein, SLR4, also increased in abundance throughout biofilm development from approximately 2% abundance in 24 h biofilms to 7.5% abundance in 72 h biofilms (Fig. 3), consistent with our previous study, which identified SLR4 as a major component of the *P. tunicata* biofilm matrix (22). The shedding of surface-layer proteins, such as SLR4, may contribute to biofilm structure and help to form a protective layer around extracellular biofilm matrix components (22, 35). The SLR4 protein was the second most abundant protein detected overall (alkaline phosphatase EAR26932 was #1) and also significantly enriched in biofilm compared to planktonic samples.

Lastly, among the top biofilm-associated proteins are hypothetical proteins whose relevance to biofilm development is unclear, including EAR27747, EAR26625, and EAR28769, which were among the top 20 biofilm-associated proteins (Table S2), and others beyond this list (e.g., EAR30269). AlphaFold modeling of EAR27747 revealed tandem bacterial immunoglobulin-like (BIg) domains similar to the structure of the invasin and intimin family of adhesins (36) (Fig. S1). The predicted structure of EAR30269, which was highly abundant in biofilms but also detected in planktonic samples, had a beta-helical fold similar to the *S. pneumoniae* surface adhesin, PfbA (37) (Fig. S1). Thus, in addition to pili, *P. tunicata* appears to produce a variety of surface adhesin proteins involved in biofilm development, most of which are uncharacterized.

### Structural modeling predicts EAR30327 is a Ca2^+^-dependent adhesin

We further investigated the top identified biofilm-associated protein, EAR30327, an uncharacterized protein of 1,600 amino acids (aa) in length annotated as “Ig-like domain-containing protein.” BLAST searches of EAR30327 identified homologs in only six additional species (all members of *Pseudoalteromonas*) with >50% coverage and *E-*value < 0.001 (Fig. S2). EAR30327’s closest identified relative was an orthologous protein in *P. ulvae*, an organism also known to colonize the marine host, *Ulva lactuca* (38) (Fig. S2). As all identified EAR30327 homologs were also uncharacterized proteins, EAR30327 is a distinct protein of unknown function that is conserved in multiple species of *Pseudoalteromonas*.

**Fig 4:**
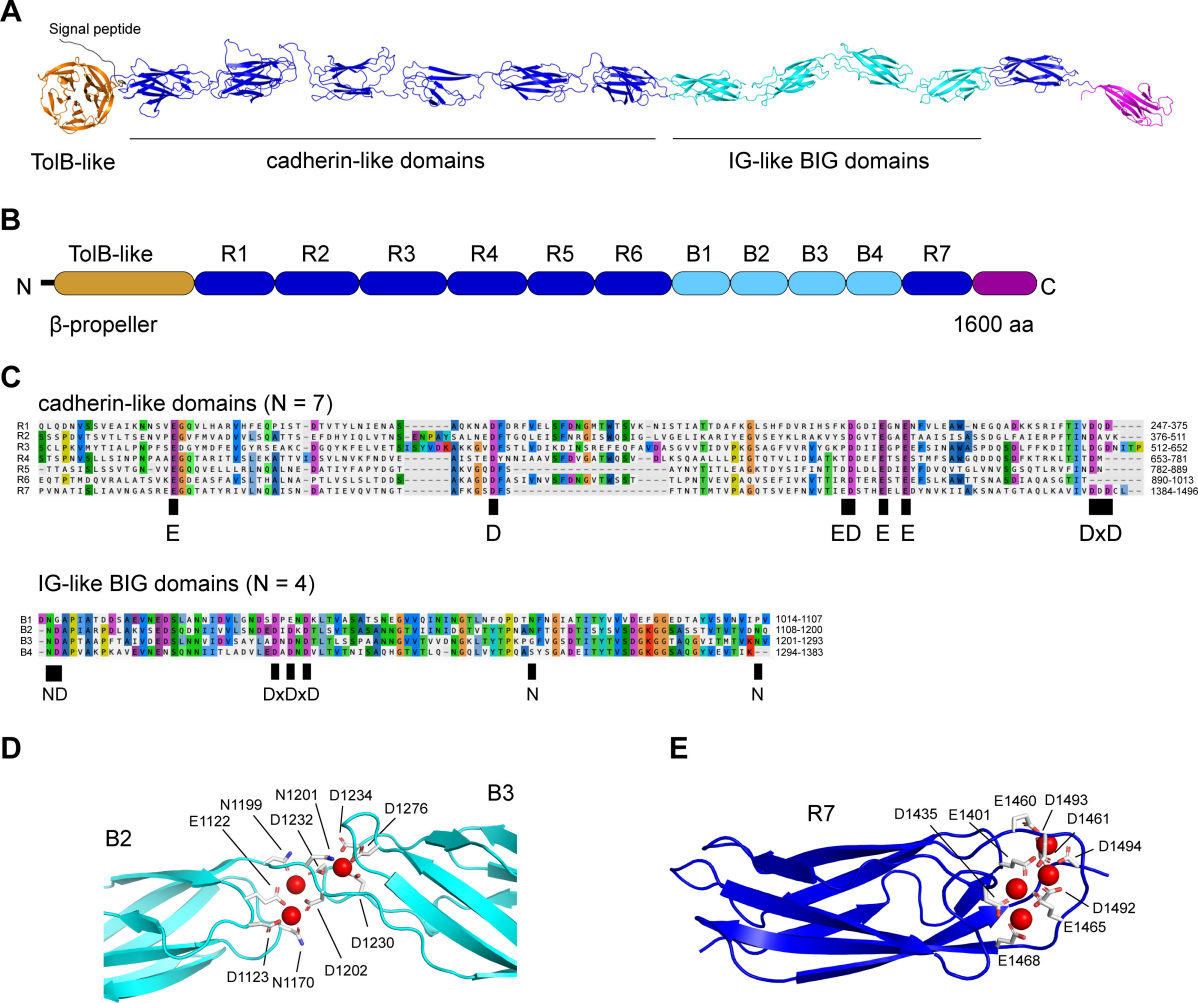
Sequence and structural analysis predicts EAR30327 (BapP) as a Ca^2+^-dependent outer membrane adhesin. (**A**) AlphaFold model of BapP colored by its domain architecture shown in panel **B**. The predicted structure of BapP consists of an N-terminal Sec/SPI signal peptide (amino acids 1–20), an N-terminal five-bladed propeller, and 12 tandem beta-sandwich repeats. The beta-sandwich repeats can be subdivided into three classes based on alignments shown in panel **C**. (**D** and **E**) Predicted Ca^2+^-binding sites (Ca^2+^ ions in red) in B2, B3, and R7 domains based on AlphaFold and AlphaFill modeling (see Materials and Methods). Key Ca^2+^-binding residues are also depicted in the alignments in panel **C**.

To gain insights into the function of EAR30327, we applied AlphaFold (39) to predict its structure (Fig. 4A; Fig. S3). The predicted structure of EAR30327 has an N-terminal domain (226 aa) with a five-bladed β-propeller fold, followed by 12 immunoglobulin-like β-sandwich folds (Fig. 4A). The 12 β-sandwich repeats can be subdivided into three classes of internal repeats (Fig. 4B and C). The first six repeats (R1-R6) and a seventh repeat (R7) found closer to the C-terminus are 108–141 aa in length and adopt a cadherin-like Greek-key structure that possesses matches to the Calx-β PFAM (PF03160) domain. A second class of shorter repeats (90–94 aa) match the BIg 9 PFAM family (PF17963) (Fig. 4B and C).

The structural architecture of EAR30327 resembles that of other biofilm-associated protein (Bap) adhesins (40–42). For instance, tandem cadherin-like domains are present in the giant RTX adhesin, SiiE, from *Salmonella enterica* (43) and also the large secreted proteins (e.g., CabD and CabC) from the marine bacterium, *Saccharophagus degradans*, which mediate Ca^2+^-dependent homophilic and heterophilic interactions (44) and have carbohydrate-binding activity (45). Calx-β like domains have also been identified in the large RTX adhesin protein, LapA (46). We, therefore, aligned EAR30327 to these other adhesins to further examine potential similarities. The N-terminal beta-propeller domain of EAR30327 did not align to the N-terminal domains of other known adhesins and appears to be a unique feature of BapP. The tandem β-sandwich repeats of EAR30327, however, aligned with similar regions of other adhesins, including LapA and CabD, with significant *E*-values and identities exceeding 30% (Fig. S4).

Within the EAR30327 sequence, we identified matches to putative Ca^2+^-binding motifs identified previously in bacterial cadherin-like domains (47), including DxD motifs, which were found in both the cadherin-like and BIg repeats (Fig. 4C). Using AlphaFold3 (48) in combination with AlphaFill (49), we modeled EAR30327’s interactions with Ca^2+^ ions (Fig. 4D and E). Numerous Ca^2+^ binding sites were predicted in the linker regions between consecutive β-sandwich domains (Fig. 4D and E), which is a common mechanism also found in Ca^2+^-dependent RTX adhesins (40, 50). The binding of Ca^2+^ ions to similar regions of RTX adhesins is thought to enable rigidification of their extender domains, allowing them to project outward to reach their targets (40, 50).

Thus, together, the sequence and structural modeling results strongly suggest a Ca^2+^-binding adhesin function for *P. tunicata* EAR30327. We, therefore, designated this protein as *Pseudoalteromonas tunicata* biofilm adhesin protein or BapP.

### BapP is required for proper biofilm formation and surface adhesion

**Fig 5:**
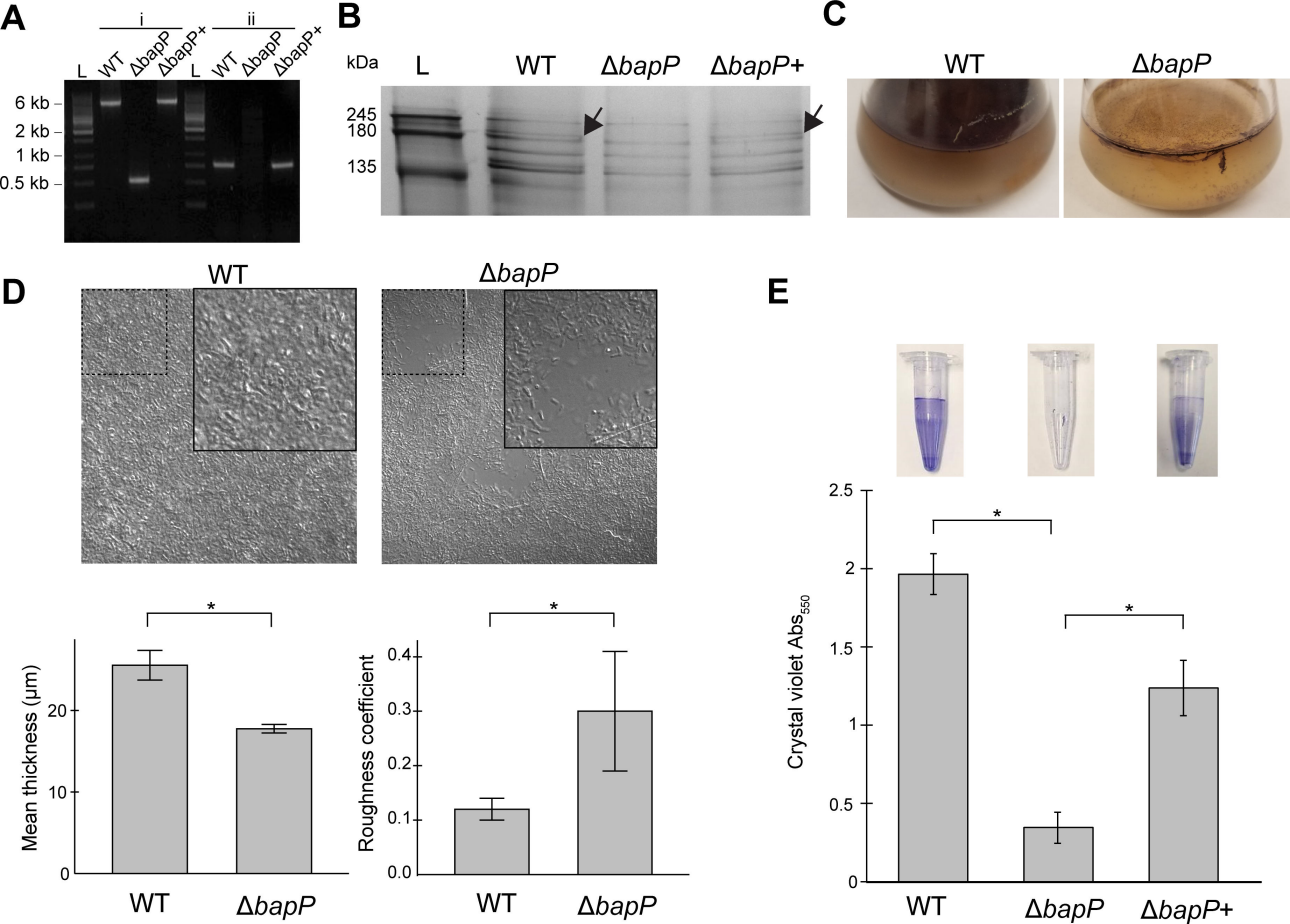
Loss of BapP reduces biofilm formation and surface adhesion. (**A**) Verification of *P. tunicata* strains by PCR amplification. Lanes 2–4 marked “i” are PCR products amplified with primers JC563 and JC564. An amplicon of 510 bp was expected in *ΔbapP* mutant, and a 5301 bp fragment was present in both wild-type and Δ*bapP*+ strains. Lanes 6–8 marked “ii” are PCR products amplified with primers JC563 and JC565. A 722 bp fragment was present in wild type and Δ*bapP*+ but absent in the Δ*bapP* strain due to the absence of the JC565 binding site. (**B**) Silver stain SDS-PAGE gel of supernatant containing extracellular proteins. As indicated by arrows, a unique band was observed in WT and rescue (Δ*bapP*+) supernatants and missing in Δ*bapP*. The band was excised from the gel and identified as BapP by liquid chromatography tandem mass spectrometry. See Fig. S5 for the original, full-size gel. (**C**) Pellicle biofilms of WT versus Δ*bapP* formed by 72 h incubation in marine broth under static conditions. (**D**) Representative confocal microscopy images of 72 h pellicle biofilms (63× magnification). (**E**) Crystal violet assays of WT, Δ*bapP*, and rescue (Δ*bapP*+) strains cultured for 24 h in centrifuge tubes containing marine broth. 95% confidence intervals are shown as error bars. The asterisks indicate significant differences in biofilm formation between WT and Δ*bapP* and between Δ*bapP* and Δ*bapP+* (*P*_adj_ < 0.01, two-tailed *t*-test, Bonferroni correction, *n* = 3 biological replicates per condition and *n* = 5 technical replicates per biological replicate).

To test the predicted function of BapP as a biofilm adhesin, we generated a Δ*bapP* knockout mutant and verified it by PCR (Fig. 5A) and sequencing of amplified PCR product from its genomic DNA (see Materials and Methods). A silver-stained SDS-PAGE gel of WT supernatants revealed a distinct band that matched BapP by LC-MS/MS analysis, and this band was absent in the Δ*bapP* strain, again confirming successful deletion of the whole *bapP* gene (Fig. 5B). The apparent molecular weight (MW) of BapP in the gel (~190 kDa) is greater than its predicted MW of 172 kDa, indicating that its migration may be affected by post-translational modifications or protein interactions. Given the difficulty in resolving individual bands in SDS-PAGE analysis of cellular protein, we examined previous shotgun proteomic data for cellular and extracellular fractions of *P. tunicata* (22, 51). BapP was detected in both the cellular (20 normalized spectral counts) and extracellular fractions (60 normalized spectral counts). Together, these data suggest that BapP is at least partially released extracellularly or, alternatively, that it is loosely associated with the cell surface. This is consistent with the biology of non-fimbrial adhesins involved in biofilm formation, which are secreted by the T1SS and loosely attached to the cell surface and often released into the extracellular space (41).

We cultured pellicle biofilms and examined their morphology over a 24–72 h period. As seen after 72 h, the WT formed robust pellicle biofilms with high cell density, whereas the Δ*bapP* mutant formed thin fragile biofilms with a loss of cohesion between cells (Fig. 5C). Regions of the Δ*bapP* biofilm were also visibly detached from the walls of the glass flask. Similarly, confocal microscopy revealed a dense and homogeneous layer of cells in WT biofilms, whereas Δ*bapP* had a fragmented and heterogeneous biofilm (Fig. 5D). Confocal Z-stack image analysis revealed that the WT biofilm was 44% thicker than the Δ*bapP* biofilm (*P* = 4.3 x 10^−7^) (Fig. 5D). Δ*bapP* biofilms also had a larger roughness coefficient (~2.5 times that of WT, *P* = 0.001), indicating a greater degree of irregularity (Fig. 5D).

To examine the reduced biofilm-forming capability of the Δ*bapP* mutant quantitatively, we performed crystal violet biofilm assays using centrifuge tubes. The WT formed robust biofilms on these tubes within a 24 h period, reaching a maximum Abs_550_ of 2.0 units, whereas there was a significant (5.7-fold) reduction in biofilm formed in the Δ*bapP* mutant (*P* = 8.6 × 10^−19^; Fig. 5E). To verify the genotypes, we generated a rescue mutant by replacing the in-frame Δ*bapP* (ATGGGATCCTAA) at its native locus with the wild-type *bapP* gene in the genome of the Δ*bapP* strain, which was then verified by PCR amplification (Fig. 5A). The Δ*bapP*+ rescue mutant successfully restored biofilm formation in the crystal violet assay (Fig. 5E). The BapP band also reappeared in the supernatant of the Δ*bapP*+ strain, confirming successful mutant rescue (Fig. 5B; Fig. S5). We also confirmed the identity of this band as BapP by mass-spectrometry analysis.

### Ca^2+^-dependent BapP-mediated biofilm formation

**Fig 6:**
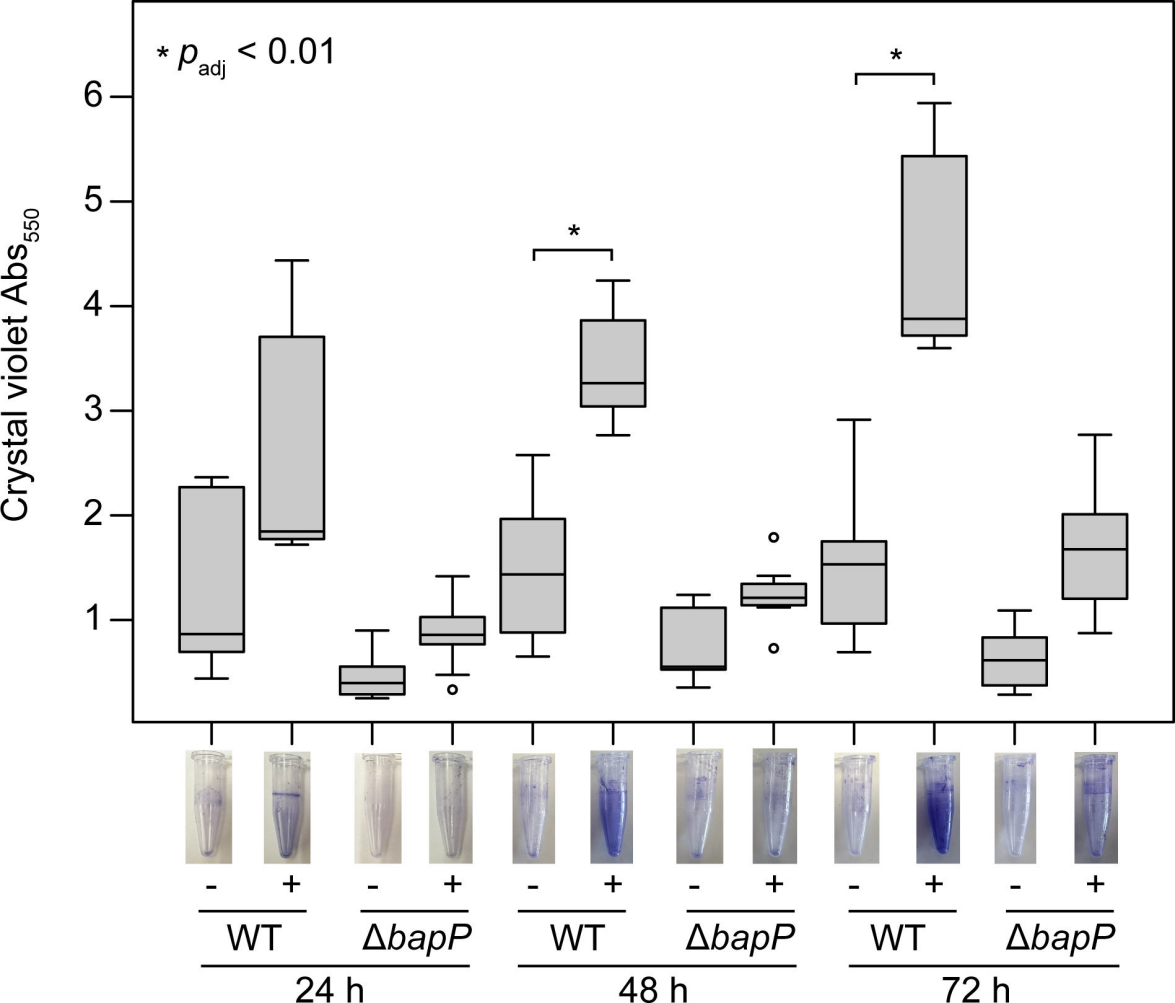
Quantification of the effect of Ca^2+^ addition on biofilm formation. Crystal violet biofilm assays were performed by culturing WT and Δ*bapP* cells in CM media over 24, 48, and 72 h periods with (+) and without (−) the addition of 1.8 g/L (16.2 mM) of CaCl_2_. The boxplots depict the distributions of Abs_550_ readings of cells attached to the walls of centrifuge tubes (*n* = 6 replicates per sample). In the WT strain, when BapP is present, the addition of Ca^2+^ is associated with a significant (*P*_adj_ < 0.01, two-tailed *t*-test with Bonferroni correction) increase in biofilm levels, but this is not observed in the Δ*bapP* strain. Boxplots show the lower quartile, median, and upper quartile of the data, with whiskers extending to 1.5 times the interquartile range (IQR) above the third quartile or below the first quartile. Images of representative tubes are shown for each condition.

Given the presence of numerous putative Ca^2+^-binding sites in BapP (Fig. 4) and the established role of Ca^2+^ in RTX biofilm adhesins (40, 50), we investigated the influence of Ca^2+^ on biofilm formation in the WT versus Δ*bapP* strain. Increased concentrations of the calcium chelating agent, EDTA, strongly inhibited WT biofilm formation (e.g., from Abs_550_ of 1.5 units without EDTA to 0.05 units with 10 mM EDTA added, *P* < 0.001) (Fig. S6). EDTA also reduced Δ*bapP* biofilm formation (e.g., 0.70 to 0.04 units for the same comparison, *P* < 0.001) but to a lesser extent, as Δ*bapP* biofilms were already significantly reduced compared to WT cells at the 24 h time point (Fig. S6). To control the CaCl_2_ concentration, we used non-marine complex media without added calcium (“CM media”) as described in Materials and Methods. Crystal violet assays were then performed with and without the addition of 1.8 g/L CaCl_2_ (16.2 mM) added to the CM media, which is equivalent to the calcium concentration of Difco marine broth 2216. In the WT strain, we observed significant increases in biofilm formation in the presence of added CaCl_2_, with a pronounced effect at later time points (Fig. 6). At the 72 h time point, crystal violet Abs_550_ readings increased from a mean of 1.5 units (no added CaCl_2_) to a mean of 4.4 units (1.8 g/L added CaCl_2_) (*P*_adj_ = 1.8 × 10^−5^) (Fig. 6). By comparison, in the Δ*bapP* strain, the addition of Ca^2+^ only increased Abs_550_ readings from 0.6 to 1.7 units (N.S., *P*_adj_ > 0.01) (Fig. 6). The weak residual Ca^2+^-dependent increase in biofilm formation in the Δ*bapP* mutant may be due to the activities of additional Ca^2+^-dependent biofilm adhesins in *P. tunicata* beyond BapP (e.g., EAR26681, which shows partial similarity to the repetitive region of BapP, Fig. S7).

To account for the possibility that Δ*bapP* had a growth defect, we measured the OD_600_ of all cells in the centrifuge tubes, including the unattached cells in the surrounding media (Fig. S8). Despite the WT showing increased biofilm formation based on crystal violet assays, with the addition of CaCl_2_, the total cell concentration increased in both the WT and Δ*bapP* strains, suggesting a general impact of Ca^2+^ on growth (Fig. S8). For example, at the 72 h time point, the OD_600_ of the WT increased from 0.6 (no added CaCl_2_) to 1.3 units (1.8 g/L added CaCl_2_/L) (*P* = 0.016), and the OD_600_ of the Δ*bapP* strain increased from 0.85 (no added CaCl_2_) to 1.6 units (1.8 g/L added CaCl_2_/L) (*P* = 0.035) (Fig. S8). However, the total concentration of cells in the tubes, including the media, was higher for Δ*bapP* than for the WT. This indicates that the observed effects (Fig. 6) are not due to growth artifacts, and that the majority of Δ*bapP* cells remained in solution, non-attached to the substrate.

### The BapP gene cluster encodes a putative Type 1 secretion system

**Fig 7:**
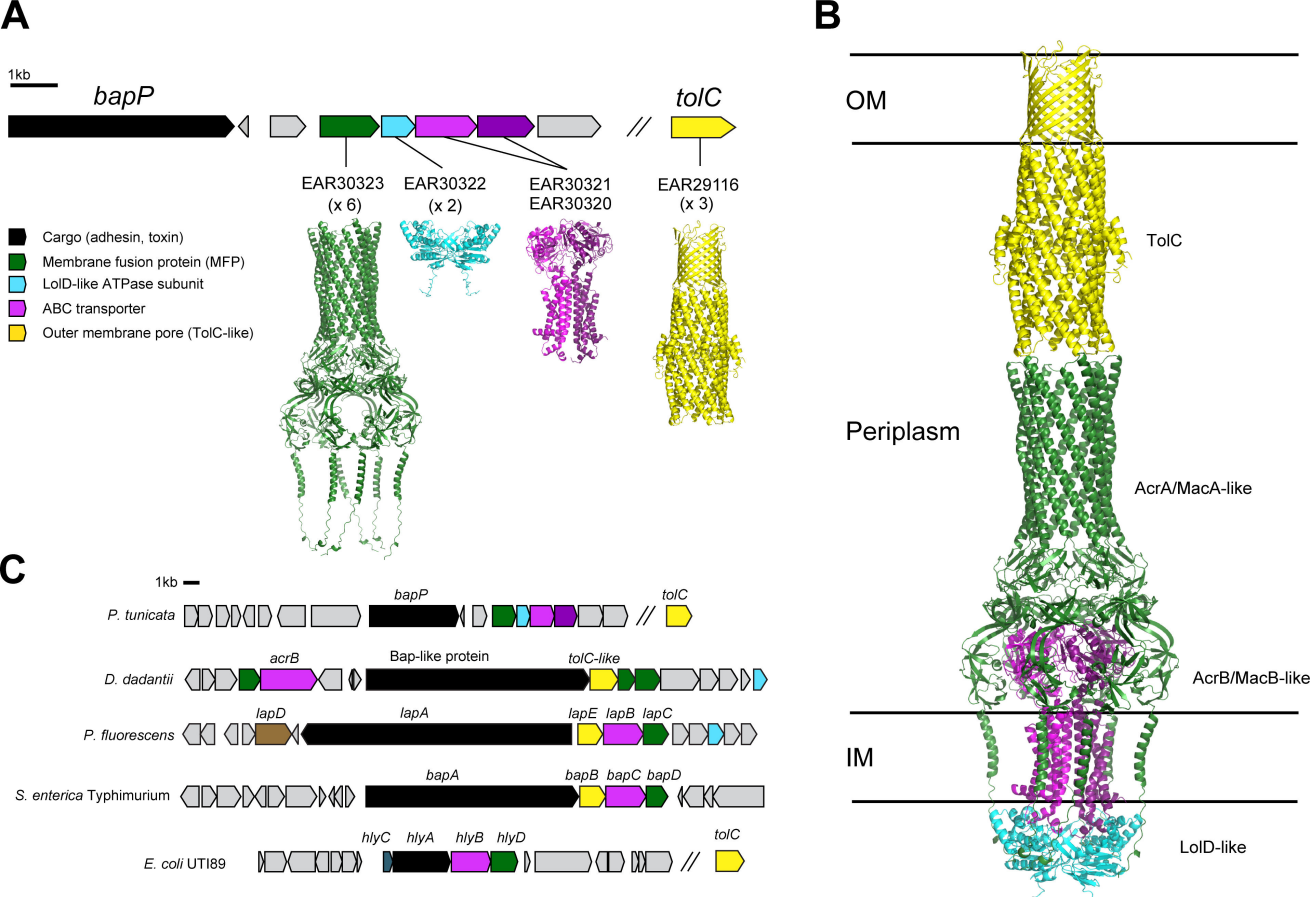
BapP is encoded next to a putative Type 1 secretion system. (**A**) *bapP* gene cluster and AlphaFold3-predicted structures of proteins encoded by the downstream genes, EAR30320-EAR30323. (**B**) AlphaFold3-predicted structure of the protein complex formed by EAR30320-EAR30323 placed alongside the predicted structure of TolC (EAR29116). EAR30320-EAR30323 were predicted as a membrane fusion protein (MFP), ABC transporter, and LolD-like ATPase subunit. As shown in panel **C**, genes encoding these components are commonly found in other T1SS-secreted adhesins and toxins, along with TolC in some cases. The genomic context similarities and structure predictions strongly suggest that BapP is encoded next to, and thus potentially secreted by, a T1SS formed by these components.

To gain further insights into the function of BapP, we examined its genomic neighborhood and used AlphaFold3 to structurally annotate adjacent protein-coding genes (Fig. 7). Immediately downstream of *bapP*, we identified four components of a T1SS, including a membrane fusion protein (MFS, EAR30323), a LolD-like inner membrane ATPase subunit (EAR30322), and tandem genes encoding ABC transporters (EAR30321 and EAR30320) (Fig. 7A). Using AlphaFold3’s multimer modeling, we predicted the complex formed by these components (Fig. 7B; Fig. S9). Remarkably, the predicted EAR30320-EAR30322 complex resembles the general structure of tripartite efflux systems (AcrAB and MacAB) and the T1SS-associated ABC transporter complex HlyBD, all of which export substrates through the TolC outer membrane pore. Figure 7B depicts our model of a secretion system formed by the EAR30320-EAR30323 complex interacting with the TolC protein of *P. tunicata* (EAR29116).

Figure 7C shows the gene neighborhoods surrounding other T1SS-secreted adhesins and toxins, including *lapA* from *P. fluorescens*, *bapA* from *S. enterica*, hemolysin A (*hlyA*) from *E. coli*, and a putative adhesin in *D. dadantii*, which had detectable homology to the repetitive region of BapP. Similar to BapP, each of the T1SS-secreted proteins is encoded adjacent to MFP and ABC transporter, which form a T1SS with a TolC-like pore. *tolC*-like genes are found immediately adjacent to *P. fluorescens lapA*, *S. enterica bapA*, and the *bap*-like gene in *D. dadantii*, but they are encoded distally in *E. coli* and in *P. tunicata*.

Together, the sequence and structural modeling of BapP’s surrounding gene neighborhood suggests a possible T1SS secretion mechanism.

### BapP similarities to other adhesins and outstanding questions

Given the functional and structural similarities of BapP to other Bap adhesins, there are several outstanding questions that are worth mentioning. Although the genomic neighborhood of *P. tunicata* BapP suggests a possible T1SS mechanism, one feature that suggests a different mechanism is the predicted Sec/SPI signal peptide at the N-terminus of BapP. This predicted Sec signal would suggest that BapP is first directed to the periplasm as an intermediate, after which it is then secreted as a passenger through a separate transport mechanism. Such mechanisms include extracellular secretion through a Type II secretion system or through a beta-barrel transporter (e.g., BamA), such as those used by the Type V secretion system. Unlike adhesins secreted by the Type Vb or two-partner secretion (TPS) system, however, BapP lacks the typical beta-helical or beta-solenoid structure found in TPS-secreted adhesins (52). It is also possible that the N-terminal Sec prediction is incorrect, and that BapP indeed possesses a T1SS signal peptide. Consistent with this hypothesis, analysis of the BapP sequence by the machine-learning tool, T1SEstacker (53), identified a high-probability (100%) T1SS signal in the C-terminal 60 aa segment.

A second outstanding question concerns the structural mechanism underlying the role of BapP in biofilm formation. The LapA adhesin, like other Bap/RTX adhesins that are secreted by the T1SS, requires its own tripartite complex (LapEBC) for secretion but remains tethered to the cell surface through a cleavable N-terminal retention module (54). However, no such region was identified in BapP with structural homology to the LapA retention module, and yet, we still identified N-terminal retention modules in homologs of BapP containing similar Big_9 repeat domains (e.g., AF-A0A066UXP9 from *Vibrio fortis*) (54). Since the N-terminal domains of RTX adhesins are generally non-repetitive and hypothesized to function as outer membrane anchors (55), it is tempting to speculate that the N-terminal beta-propeller domain of BapP plays a similar role, but this needs to be experimentally tested. A second feature of RTX adhesins is the presence of extender regions, which are rigidified and strengthened by Ca^2+^ to project C-terminal binding domains to their targets (50). Likely domains playing this role in BapP are the cadherin-like and BIg domains, which are structurally homologous to those in LapA and other adhesins (50). The observed impact of calcium in modulating the activity of BapP is, therefore, potentially related to this mechanism. Calcium has also been shown to play other roles in modulating adhesin activity; however, such as that in the *Rhizobium*-adhering protein RapA2 lectin, where calcium has been shown to impact RapA2’s ability to bind acidic exopolysaccharides (56). Finally, the ligand-binding domain and exact substrate of BapP are also to be determined. Given that the ligand-binding domains of RTX adhesins tend to occur at the distal C-terminus (50), and that the C-terminal (12th) beta-sandwich domain in BapP is unique in sequence from all other repeats indicating an adaptation to a unique function, we also speculate that it may play this role in BapP.

Ultimately, as we have relied on bioinformatics to make predictions about BapP’s secretion mechanism and structure-function relationship, both must be further investigated through experimental studies. Knockout studies of different domains will be essential for examining BapP’s secretion mechanism, the nature of its Ca^2+^-dependent interactions and targets, and the function of different domains in mediating adhesion to biotic surfaces, abiotic surfaces, cell-cell adhesion, EPS and biofilm matrix, or perhaps other adhesive functions that could influence biofilm formation. Such studies will help to determine the extent to which BapP is functionally equivalent to other RTX Bap adhesins.

### Conclusion

Biofilm formation by marine bacteria is critical for marine microbial ecology and has substantial practical, industrial, and economic implications (57). In this work, we explored the proteomic determinants of biofilm development in the marine microorganism *P. tunicata*, which serves ecologically important roles in marine biofilms and possesses a mostly uncharacterized proteome containing an abundance of hypothetical proteins. Comparative shotgun proteomics detected hundreds of proteins with increased abundance in *P. tunicata* biofilms, many of which are either uncharacterized or have currently unknown roles in biofilm development. Longitudinal analysis over a 24–72 h period also revealed temporal patterns of protein expression, reflecting changes in biofilm function in different stages of biofilm development. Through in-depth proteomic analysis of biofilms, we were able to overcome the technical challenges of separating proteins from what can be highly complex and challenging samples to work with.

In addition to identifying hundreds of biofilm-associated proteins and providing a unique hypothesis-generating proteomic data set for future studies, our work led to the discovery of a Ca^2+^-dependent adhesin (BapP) in *P. tunicata* and related species, which we confirmed experimentally as playing a key role in biofilm formation. BapP appears to share structural and functional similarities with other biofilm adhesin proteins (e.g., LapA) but is distantly related in sequence and also possesses entirely unique domains (a predicted N-terminal beta-propeller). The BapP gene cluster also suggests a possible T1SS mechanism similar to other RTX adhesins, establishing BapP as a new member of the broad class of Bap adhesins.

## MATERIALS AND METHODS

### Culturing of *P. tunicata* samples throughout planktonic to biofilm development

A frozen stock of *P. tunicata* strain D2 was streaked on Difco marine agar 2216 and incubated for 48 h at room temperature. An overnight culture was made by inoculating a colony into 4 mL of marine broth, which was incubated overnight at 24°C with 120 rpm. Eight subcultures were made in Erlenmeyer flasks by transferring 1 mL (OD_600_ = 1.3) of the overnight culture into 100 mL of marine broth. The flasks were incubated for 8 h at 24°C with gentle shaking (100 rpm). After 8 h incubation, two of these cultures were used as planktonic shaking cultures and considered “T0” samples. The six remaining flasks were used to generate pellicle (air-liquid interface) biofilms by incubating these flasks statically at room temperature for 3 days. Samples were collected at 24, 48, and 72 h of incubation. Duplicate samples were collected from each time point, and they were pooled in one tube. Biofilm samples were collected from the center and the edge of the biofilm in addition to surrounding media below the biofilm (planktonic static samples). The entire procedure was repeated three times to provide biological replicates. Thus, a total of 30 samples were collected from four different time points (*T*) as follows: *T*0 = [(planktonic 8 h shaking) (*n* = 3)]; *T*24 = [24 biofilm sample centers (*n* = 3), biofilm sample edge (*n* = 3), planktonic static (*n* = 3)]; *T*48 = [48 biofilm sample centers (*n* = 3), biofilm sample edge (*n* = 3), planktonic static (*n* = 3)]; *T*72 = [72 biofilm sample centers (*n* = 3), biofilm sample edge (*n* = 3), planktonic static (*n* = 3)].

A method used by Park et al. (58) with some modifications was followed for sample processing. To collect planktonic shaking samples, 7.5 mL was aspirated from each of two flasks using a sterile serological pipette, yielding a total volume of 15 mL. The planktonic shaking cultures were then concentrated to a volume of 2.5–3 mL using a Vivaspin 20 centrifugal filtration unit with a 3 kDa cutoff operated in a swing bucket at 4,000 ×*g* for 140–160 min at 4°C. The concentrate (2.5–3 mL) was washed using the same Vivaspin 20 unit with 10 mL of cold Tris-HCl buffer (0.1 M, pH 8.3). This washing step was performed with a fixed-angle rotor at 6,000 ×*g* for 10 min at 4°C. A final volume of 500 µL of concentrate was collected, and the samples were then frozen at −20°C.

Pellicle biofilm samples were collected using a clean round coverslip (Fisher brand, No. 2, 0.25 mm thick, size 18 mm) attached to a custom-designed tool composed of a spring and a metal rod shown in Fig. 1, which was cleaned with water and alcohol after each use. Biofilm samples (18 mm diameter) were collected from the edge and the biofilm’s center. The coverslips containing biofilm were placed in a clean tube containing 5 mL of cold Tris-HCl buffer (0.1 M, pH 8.3). Biofilm samples were washed off the surface of the coverslip tube by vortexing gently until the biofilm was completely separated from the coverslip and transferred to clean tubes. The biofilm suspension was centrifuged once at 12,000 × *g* for 10 min at 4°C. The supernatant was discarded. The pellet was collected and resuspended in 500 µL of cold Tris-HCl (pH 8.3) and vortexed until all the pellet was resuspended. The samples were frozen at −20°C. Planktonic static samples were also collected from each biofilm flask after the biofilm samples were taken and prepared using the same protocol as described above for planktonic shaking samples.

### Shotgun proteomics

#### Sample processing for liquid chromatography tandem mass spectrometry

Prior to liquid chromatography tandem mass spectrometry (LC-MS/MS) analysis, samples were processed for protein extraction and quantification. Three rounds of freeze/thaw cycles were performed using 1 L of liquid nitrogen for 30 s, followed by transfer to a room temperature water bath. Samples (990 µL) were then treated with 10 µL of protease inhibitor cocktail FOCUS ProteaseArrest (G-Biosciences) and kept on ice. A 3 mm sonicator tip (Qsonica Sonicator) was used to sonicate the samples, which were placed in 2 mL round-bottom Eppendorf tubes. Sonication was performed in four cycles on ice, with each cycle consisting of 30 s at 30% amplitude, followed by 60 s of cooling time between sessions. The sonicated samples were centrifuged (6,000 ×*g*, 10 min, 4°C) to remove cellular debris, and the supernatants were collected. The samples were stored at −20°C for further processing. To perform LC-MS/MS, at least 50 µg of protein sample was collected for each sample. The protein concentration in the lysates was measured using the Bradford assay (59).

#### Proteomics

Ten micrograms of samples was reduced with 10 mM dithiothreitol (Sigma-Aldrich, Missouri, USA) alkylated with 20 mM iodoacetamide (Sigma-Aldrich, Missouri, USA), acetone precipitated, and digested overnight with MS grade trypsin (Promega, Wisconsin, USA). Digested samples were lyophilized, then resuspended in 0.2% trifluoroacetic acid and desalted using a homemade C18 zip tip (resin: Empore, 2215-C18 [octadecyl]). The C18 desalted samples were resuspended in 18 µL buffer A (0.1% FA buffer, pH 2.7). Six microliters of each sample was injected into the *tims*TOF Pro (Bruker Daltronics, Bremen, Germany) using nanoflow liquid chromatography using a Bruker NanoElute chromatography system (Bruker Daltronics, Bremen, Germany). Liquid chromatography was performed at a constant flow of 400 µL/min and a 15 cm reversed-phased column with a 75 µm inner diameter, packed with Reprosil C18 (PepSEP, Bruker, Germany). Mobile phase A was 0.1% formic acid, and mobile phase B was 99.9% acetonitrile and 0.1% formic acid. The *tims*TOF Pro was outfitted with a CaptiveSpray source (Bruker Daltronics, Bremen, Germany) operated in parallel accumulation-serial fragmentation mode. MS and MS/MS scans were limited to 100 to 1,700 *m*/*z*, and a polygon filter was applied to the *m*/*z* and ion mobility dimensions to select for multiple charged ions most likely to be peptide precursors. Collision energy was applied as a function of ion mobility using a linear regression with the following parameter settings: 0.85 V·s/cm² at 27 eV and 1.30 V·s/cm² at 45 eV. TIMS voltage was calibrated using ions from the Agilent tune mix (*m*/*z* 622, 922, 1222). Active exclusion of MS/MS scans was enabled with a setting of 0.40 min. Quadrupole isolation was set to 2 *m*/*z* for ions with *m*/*z* less than 700 and 3.0 *m*/*z* for ions with *m*/*z* greater than 800. All MS experiments were completed at the Bioinformatics Solutions, Inc. MS lab (Waterloo, Ontario, Canada).

MS raw files were processed using PEAKS XPro (v10.6, Bioinformatics Solutions, Inc., Ontario, Canada). The data were searched against a custom database containing the *P. tunicata* proteome. Precursor ion mass error tolerance was set to 20 ppm, and fragment ion mass error tolerance was set to 0.05 Da. Semi-specific cleavage with trypsin was selected with a maximum of two missed cleavages. A fixed modification of carbamidomethylation (+57.02 Da) on cysteine residues was specified. Variable modifications of deamidation (+0.98 Da) on asparagine and glutamine, as well as oxidation (15.99 Da) on methionine, were specified. The false discovery rate threshold was set to 1% for the database search.

Normalized protein abundance calculated by Peaks was further converted to proportions of overall counts using R v4.3.3. Heatmaps of protein abundance were generated using pheatmap (https://github.com/raivokolde/pheatmap) with row normalization applied using the scale="row" option. PCA was performed using the “prcomp” function in R. Differential analysis of protein abundance was performed using two-tailed *t*-tests with *P* values adjusted for multiple hypotheses using p.adjust with the Benjamini-Hochberg method (60). Differentially abundant proteins were identified as those with log_2_FC values > 0.5 and *q-*values < 0.01.

### Bioinformatic analysis of EAR30327

EAR30327 (BapP) homologs were identified by BLAST analysis of the NCBI nr database on 1 July 2024 using an *E*-value threshold of 0.001 and a query coverage threshold of 50%. A phylogenetic tree of BapP and close homologs from other *Pseudoalteromonas* species was produced using PhyML v3.1 (61) using 1018 sites and the LG model with four rate classes.

Structural modeling of EAR30327 was initially performed using ColabFold’s implementation of AlphaFold2 (39, 62) and later refined using AlphaFold3 (48). Structural models were then edited to obtain a linear domain representation for improved visualization in PyMOL v3.0 (https://pymol.org/) and connective loops closed within MODELLER (63). Internal sequence repeats based on the structural model were further aligned using MUSCLE (64) and further edited and visualized using AliView (65). To allocate metal-binding sites, AlphaFill (49) was used to identify potential Ca^2+^-binding sites based on homology to other Ca^2+^-binding template structures, and identified Ca^2+^-binding domains were then submitted individually or as pairs to AlphaFold3 for placing high-confidence calcium ions (pLDDT > 75). As in the context of the full-length protein sequence, only low-confidence sites could be predicted (pLDDT < 25).

Gene neighborhoods surrounding BapP and other selected proteins (T1SS-secreted adhesins and toxins) were visualized and explored using the AnnoView browser (66) and AnnoTree database of microbial genome annotations (67). Structural and functional annotations were performed using a combination of AlphaFold3 (48) predictions and existing sequence-based annotations (CDD [68], Interpro [69]) from the NCBI and Uniprot databases. Based on the stoichiometry of components in AcrAB-TolC (70) and MacAB-TolC (71) systems, an AlphaFold3 model was generated for a protein complex composed of six monomers of the putative membrane fusion protein EAR30323, two monomers of the LolD-like subunit EAR30322, and a heterodimer of the ABC transporter proteins EAR30320 and EAR30321. The resulting complex was then manually placed alongside the predicted trimer structure of *P. tunicata* TolC (EAR29116).

### Construction of Δ*bapP* and rescue (Δ*bapP*+) plasmids

#### Bacterial strains, plasmids, and growth media

Bacterial strains and plasmids used in this study are derived from earlier work (4, 72, 73) and listed in Table S6. *Escherichia coli* strains were grown at 37°C in Luria-Bertani medium (1.0% tryptone, 0.5% yeast extract and 0.5% NaCl). *P. tunicata* was grown in Difco marine broth 2216. Enzymes were obtained from New England Biolabs (NEB). Antibiotics were used at the following final concentrations: kanamycin, 50 µg/mL for *E. coli* or 100 µg/mL for *P. tunicata*; chloramphenicol, 20 µg/mL.

#### Plasmid construction

Q5 high-fidelity DNA polymerase (NEB) was used for plasmid construction and confirming constructs. The upstream region (1,469 bp) of the *bapP* gene and 23 bp downstream of the gene were PCR-amplified using oligos JC559 and JC560 (Table S7; Fig. S10), and its downstream region (1,690 bp) and 20 bp upstream of the ORF and start codon (ATG) were obtained using primers JC561 and JC562. Both DNA fragments were gel-purified and pooled in equal amounts as PCR template using JC559 and JC562. The PCR product was digested with EcoRI-XbaI, and then inserted into the same sites in pK19mobsacB, yielding plasmid pJC296.

A DNA fragment (6818 bp) containing *bapP* ORF, upstream and downstream regions (989 and 1,014 bp, respectively) of the gene were PCR-amplified using oligos JC679 and JC680 (Table S7; Fig. S10). After gel purification, the amplicon was restricted with EcoRI-XabI, and then cloned into the same sites in pK19mobsacB to obtain plasmid pJC302. The plasmid constructions were verified by restriction enzyme mapping.

#### Construction of *P. tunicata* strains

Plasmid pJC296 was conjugated into *P. tunicata* D2 with the helper plasmid, pRK600 (74). Single cross-over recombination of the pJC296 into *P. tunicata* genome was selected on marine agar with kanamycin. A single colony was streak-purified on a fresh plate. A colony was grown in Difco marine broth 2216, diluted serially, and plated on marine agar plates containing 5% sucrose. Resulting clones were tested for kanamycin sensitivity (double cross-over and loss of plasmid backbone). Genomic DNA was isolated from the Kan^S^ clones. DNA fragments were PCR-amplified using primer pairs of JC563-JC565 and JC563-JC564. The products were resolved with 1.5% TAE agarose gel. The amplicon generated with oligos JC563 and 564 was also Sanger-sequenced at The Centre for Applied Genomics (Toronto, Ontario).

In order to rescue *bapP* ORF in the in-frame deletion Δ*bapP* mutant, plasmid pJC302 was transferred into the Δ*bapP* mutant strain with the plasmid pRK600. Following the selection of single cross-over recombination on marine agar with kanamycin, one transconjugant clone was streak purified on a new selection plate. A Kan^R^ colony was then incubated overnight in marine medium, diluted serially, and plated on marine agar with 5% sucrose. Resulting clones were screened for kanamycin sensitivity (*bapP* ORF inserted and loss of plasmid backbone). In order to detect the replacement of in-frame Δ*bapP* ORF in the mutant, genomic DNA was extracted, and PCR amplification was performed using primer pairs of JC563-JC565 and JC563-JC564.

### Crystal violet biofilm assays

A modified protocol based on a previous study (75) was used to perform crystal violet assays. WT, Δ*bapP*, and rescue (Δ*bapP*+) strains were grown overnight in Difco marine broth 2216 at 24°C with shaking at 120 rpm. Overnight cultures were diluted to an OD_600_ of 0.01 with Difco marine broth 2216, and 900 µL of each subculture was dispensed into regular retention 1.5 mL microcentrifuge tubes (GeneBio). The centrifuge tubes were incubated statically at room temperature for 24 h. Five replicates were made for each sample, and the entire experiment was replicated three times to ensure reproducibility. After the appropriate incubation time, the culture was discarded. The biofilm adhered to the walls of the microcentrifuge tubes was stained by adding 950 µL of 0.1% crystal violet to each microcentrifuge tube and left to stain for 15 min. Then, the stain was disposed of, and the excess stain was removed by shaking out the tube and slowly washing with water three times. The trapped water was shaken out, and the biofilm was left to dry overnight. To quantify the biofilm, the concentration of crystal violet was measured. Next, 1.1 mL of 30% acetic acid was added to each microcentrifuge tube to solubilize the crystal violet for 15 min. Then, 1.1 mL of 30% acetic acid was added to three microcentrifuge tubes to serve as a blank. The absorbance was quantified using a spectrophotometer (BioSpectrometer, Eppendorf) at 550 nm.

The same procedure described above was then used to investigate the effects of CaCl₂ on BapP biofilm formation with one modification: the overnight cultures of WT and Δ*bapP* strains were grown in complex media “CM” broth (tryptone 10 g, yeast extract 5 g, NaCl 10 g, MgSO_4_ 0.150 mg, deionized water 1 L) with five different levels of CaCl_2_ (0, 0.138, 0.277, 0.832, 1.8 g/L; 0, 1.24, 2.49, 7.50, 16.2 mM). Each culture was diluted to an OD_600_ of 0.01 with a volume of the corresponding CM broth. Then, each subculture was dispensed into microcentrifuge tubes. Three replicates were made from each subculture, and the entire experiment was replicated three times to ensure reproducibility.

### Confocal laser scanning microscopy and image analysis

Pellicle biofilm samples were collected using a clean square coverslip (Fisher brand, No. 1.5, 0.17 mm thick, size 18 × 18 mm) and the same custom-designed tool that was described earlier to collect the biofilm samples for proteomic analysis. The coverslip was immediately inverted on a clean microscopic slide, and the edges of the coverslip were sealed with a transparent nail polish to avoid biofilm dryness. Slides were examined within 1 h of preparation using a Zeiss LSM 700 confocal microscope with the differential interference contrast II condenser setting at magnifications of 40× and 63×.

To facilitate region-specific biofilm analysis, the images were divided into technical replicates using a standardized region of interest (ROI) selection approach. The full biofilm area was systematically partitioned into nine equally sized ROIs per sample to ensure spatially representative sampling. Each ROI was selected using ImageJ’s ROI Manager tool to ensure uniformity across all images. To maintain consistency, the same selection pattern was applied to both WT and mutant images. Each ROI was extracted as an individual .tiff file and analyzed separately. Quantitative biofilm analysis was performed using COMSTAT2 (76), a MATLAB-based image-processing tool. Each extracted ROI was processed independently to obtain structural metrics, including mean thickness and roughness coefficient. Image stacks were first binarized to segment biofilm regions from the background, followed by thresholding to eliminate noise. The thresholding parameters were optimized to prevent under- or over-segmentation while maintaining structural integrity. Biomass was quantified as the total biofilm volume per unit area, and thickness distribution was calculated across all optical slices to generate an average thickness measurement for each region. Maximum thickness was recorded as the largest vertical extent of the biofilm within each ROI. The roughness coefficient was derived as a measure of surface irregularity, where higher values indicated increased heterogeneity in biofilm topology. The surface-to-biovolume ratio was determined to assess biofilm expansion and structural complexity, with higher ratios indicating greater surface coverage relative to total biomass. Statistical comparisons between WT and mutant biofilms were conducted using Welch’s *t*-test to account for unequal variances between the groups. Mean and standard deviation were calculated for each biofilm parameter across the nine technical replicates. Degrees of freedom were adjusted based on variance estimates, and *P*-values were computed to determine statistical significance. A significance threshold of *P* < 0.05 was used to confirm differences in biofilm structure between WT and mutant strains. All statistical analyses were performed using Python’s SciPy package.

### SDS-PAGE

WT, Δ*bapP*, and Δ*bapP+* strains were grown overnight in 4 mL Difco marine broth 2216 at 24°C with shaking at 120 rpm. Overnight cultures were subcultured by first standardizing overnight to an OD_600_ of 1.0 with Difco marine broth 2216. The diluted overnight cultures were then subcultured to a 1:99 dilution into fresh marine broth. A total volume of 4 mL was prepared and incubated for 5 h at 24°C with shaking at 120 rpm. Each culture was centrifuged at 5,000 × *g* and 4°C for 15 min. The supernatant was transferred to a new container and centrifuged again under the same conditions. The resulting supernatant was treated with two different protease inhibitors to prevent protein degradation: 10 µL of FOCUS ProteaseArrest added to 990 µL of the sample and 10 µL/mL of 0.5 M EDTA. The samples were stored at −20°C. On the following day, samples were subjected to SDS-PAGE (10% SDS) for protein separation based on molecular weight. A 25 µL of samples was loaded into the gel. In addition, 5 µL of BLUelf prestained ladder with MW 5–245 kDa (FroggaBio) was loaded. The gel was run at 90 kV for 20 min and 140 kV for 60 min. The SDS-PAGE gel was stained using silver staining (Pierce Silver Stain for Mass Spectrometry, Thermo Scientific) following the kit protocol.

## Data Availability

Data are provided within the paper or supplemental files. Additional raw data sets used and/or analyzed during the current study are available from the corresponding author on reasonable request.

## SUPPLEMENTAL MATERIAL

Supplemental Material mbio.01069-25-s0001.pdf Fig. S1–S10 and supplemental table legends.

Supplemental Tables mbio.01069-25-s0002.xlsx Tables S1–S7.

ASM does not own the copyrights to Supplemental Material that may be linked to, or accessed through, an article. The authors have granted ASM a non-exclusive, world-wide license to publish the Supplemental Material files. Please contact the corresponding author directly for reuse.
