# Supplementary material for: Comparative proteomics of biofilm development in Pseudoalteromonas tunicata discovers a distinct family of Ca2+-dependent adhesins
Source: mBio. 2025 May 21;16(6):e01069-25. doi: 10.1128/mbio.01069-25 (PMC12153261; doi:10.1128/mbio.01069-25)
Supplement: Supplemental Material — Fig. S1–S10 and supplemental table legends. [file mbio.01069-25-s0001.pdf]

SUPPLEMENTARY MATERIAL

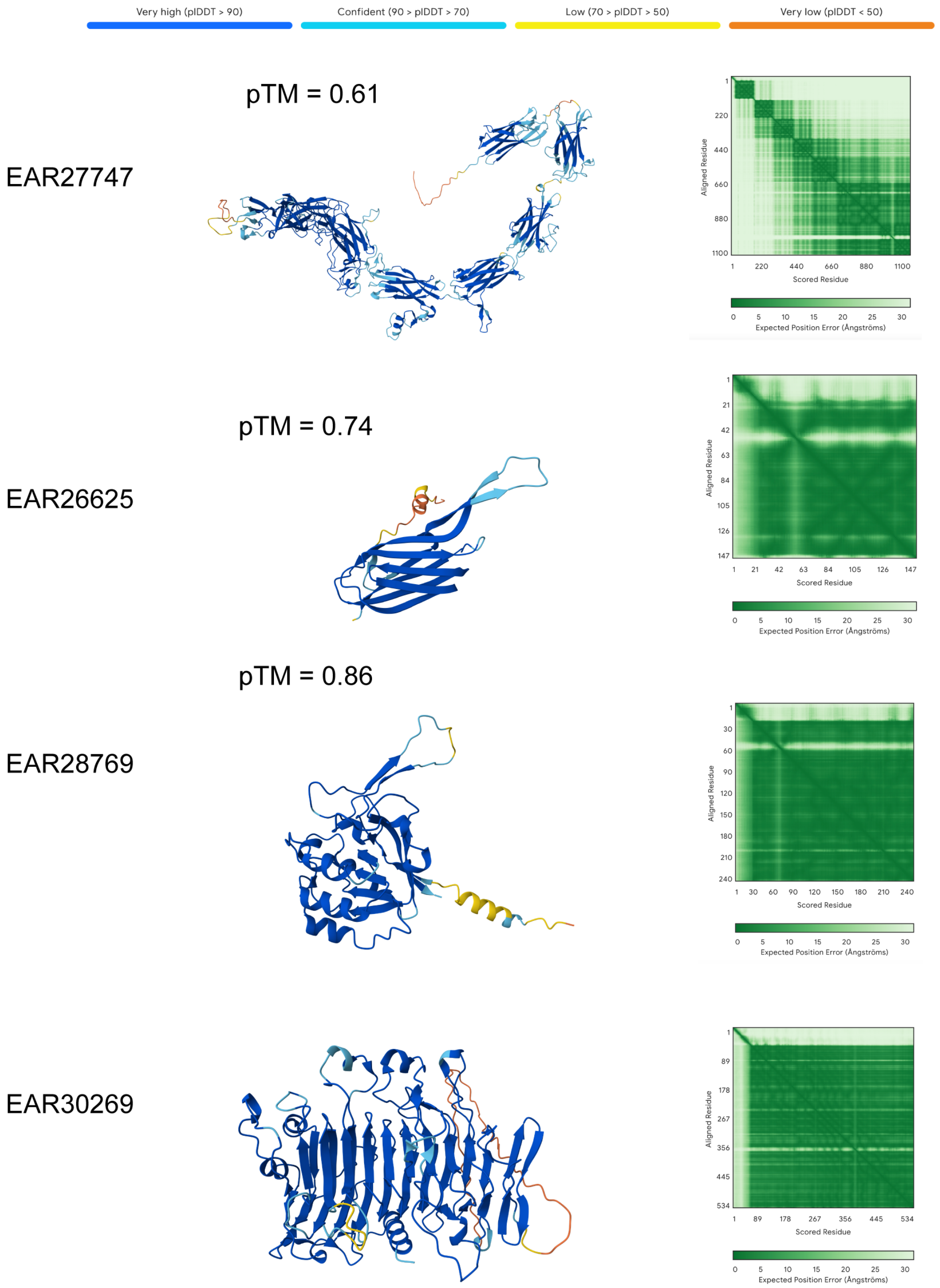

Figure S1. AlphaFold3 structural models of four *P. tunicata* proteins of unknown function with increased LC-MS/MS abundance in biofilm samples. Predicted structures of EAR27747, EAR26625, EAR28769, and EAR30269 are shown on the left, with their corresponding predicted aligned error (PAE) plots on the right. The PAE plots indicate the AlphaFold3 confidence in the relative position of residue pairs within the predicted structure.

A

Description	Total Score	Query Cover	E value	Per. ident	Acc. Len	Accession
Ig-like domain-containing protein [Pseudoalteromonas tunicata]	3231	100%	0	100	1600	WP_009836625.1
Ig-like domain-containing protein [Pseudoalteromonas tunicata]	3226	100%	0	99.81	1600	WP_305975243.1
Ig-like domain-containing protein [Pseudoalteromonas tunicata]	3221	100%	0	99.69	1600	WP_306107941.1
Ig-like domain-containing protein [Pseudoalteromonas ulvae]	2479	100%	0	75.62	1600	WP_086743690.1
Ig-like domain-containing protein [Pseudoalteromonas ulvae]	2477	100%	0	75.62	1600	WP_193331482.1
Ig-like domain-containing protein [Pseudoalteromonas spongiae]	1640	86%	3e-100	29.46	2341	WP_336436765.1
Ig-like domain-containing protein [Pseudoalteromonas spongiae]	966	86%	4e-96	28.51	2341	WP_100914984.1
Ig-like domain-containing protein [Pseudoalteromonas sp. T1lg24]	791	70%	2e-92	28.85	1660	WP_105171922.1
Ig-like domain-containing protein [Pseudoalteromonas piratica]	952	87%	1e-91	29.17	2341	WP_040136050.1
Ig-like domain-containing protein [Pseudoalteromonas sp. P1-9]	955	86%	1e-89	28.35	2341	WP_054980746.1
Ig-like domain-containing protein [Pseudoalteromonas spongiae]	953	86%	1e-89	28.58	2341	WP_010559390.1
Ig-like domain-containing protein [Pseudoalteromonas sp. MMG024]	952	86%	4e-89	28.71	2341	WP_237129147.1
Ig-like domain-containing protein [Pseudomonadota bacterium]	938	86%	9e-89	28.78	2341	MEC8327087.1
Ig-like domain-containing protein [Pseudoalteromonas sp.]	704	72%	9e-78	27.88	1538	WP_372769392.1

B

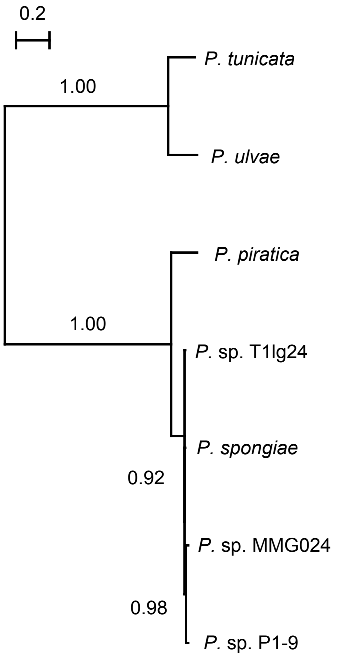

Figure S2. Identification and phylogenetic analysis of EAR30327 homologs in six additional species of *Pseudoalteromonas*. A) BLAST result using EAR30327 as a query against the full NCBI nr database. All homologs with E-values < 0.001 and query coverage > 50% are shown. B) Maximum-likelihood phylogenetic tree of EAR30327 from *P. tunicata* along with non-redundant homologs in other *Pseudoalteromonas* species. Bootstrap values are shown above the nodes.

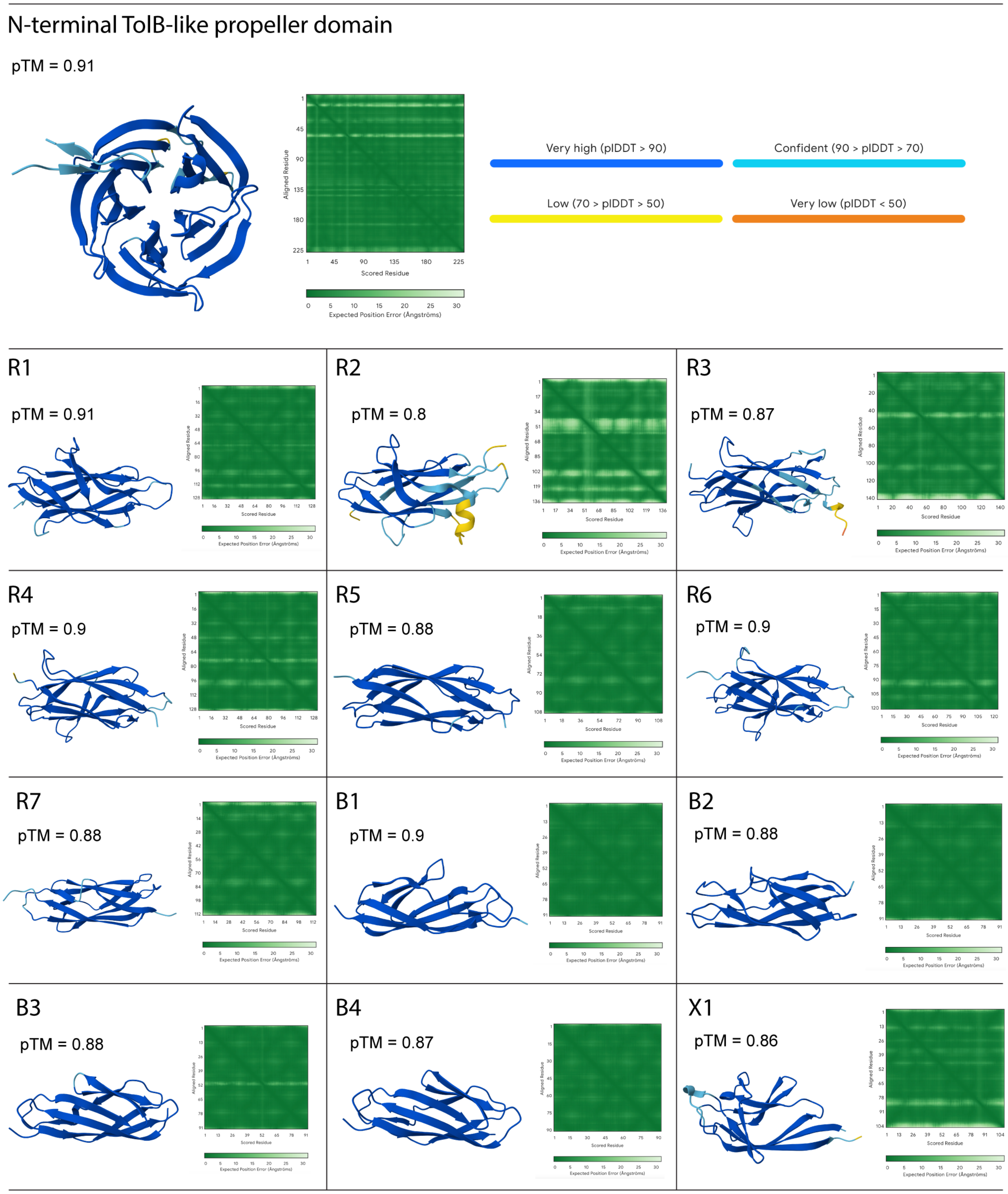

Figure S3. AlphaFold3-predicted structures of BapP domains. The predicted structure of BapP's N-terminal domain adopts a five bladed propeller fold, and the remaining 12 tandem repeat domains adopt immunoglobulin-like beta-sandwich structures. PAE plots are shown on the right of each structural model and indicate the per-residue confidence scores. pTM scores range from 0.8 to 0.91.

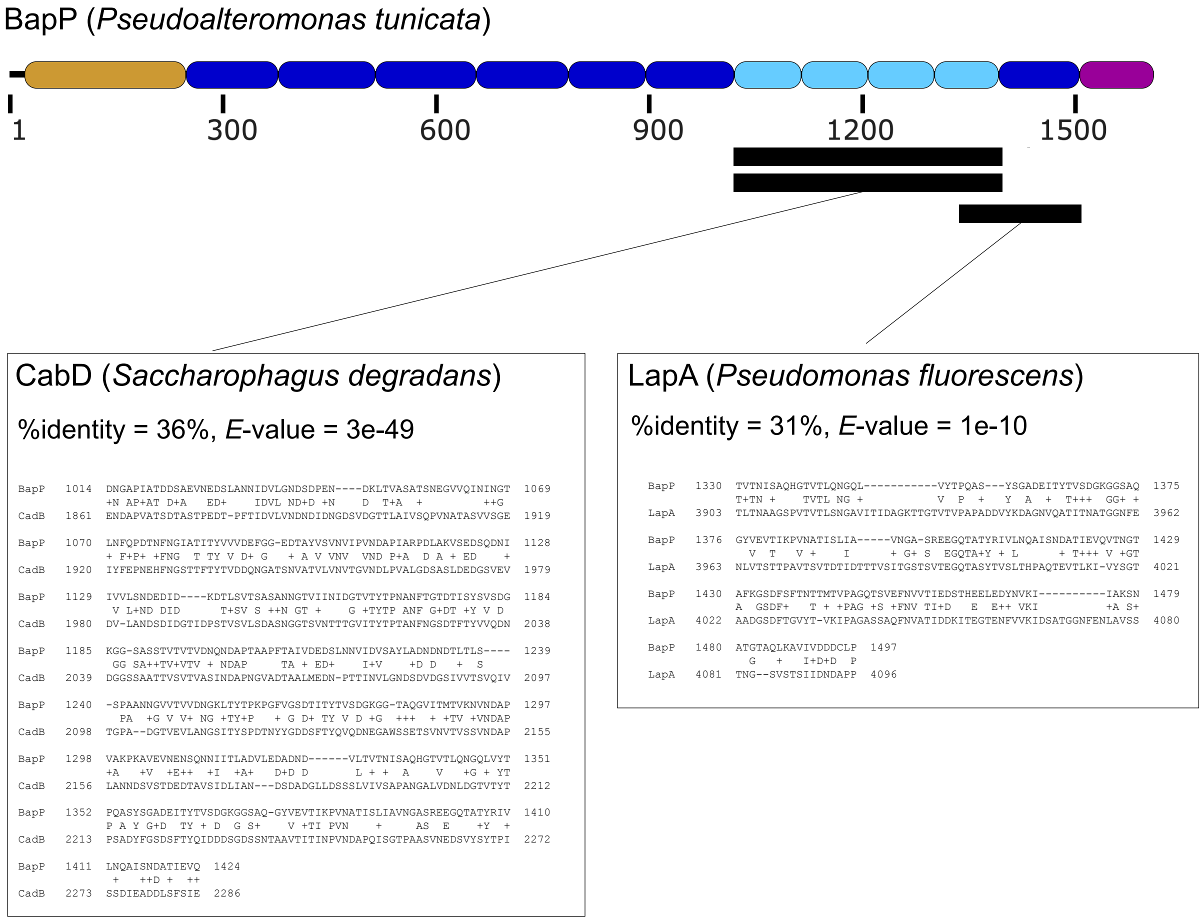

Figure S4. Partial sequence homology between a putative binding region in BapP and binding domains from other biofilm adhesins. A domain architectural model of BapP is depicted above, and two regions corresponding to significant BLAST alignments are shown below, the first of which is similar to a region from the CadB adhesin from *S. degradans*, and the second of which is similar to a region from *P. fluorescens* LapA.

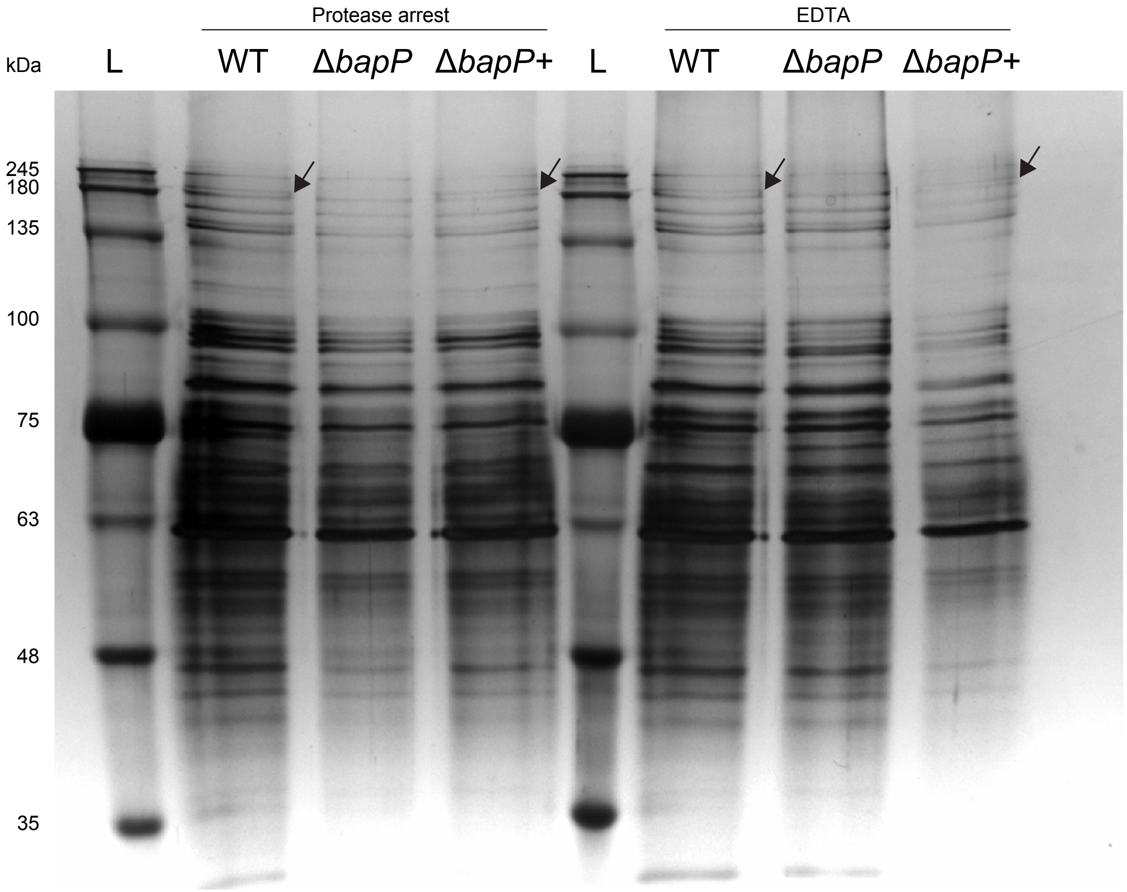

Figure S5. SDS-PAGE gel of supernatant protein collected from the WT, Δ*bapP*, and Δ*bapP*+ strains. Protease arrest and EDTA were added to minimize protein degradation. A unique band (arrow) appeared at ~180 kDa in the WT and Δ*bapP*+ strains, and not in the Δ*bapP* deletion strain, and was identified as BapP by LC-MS/MS proteomics.

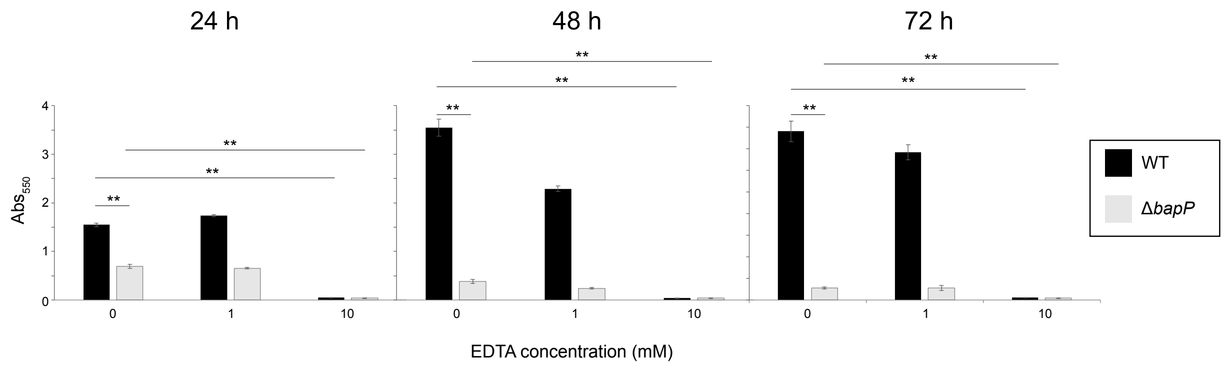

Figure S6. Impact of EDTA on WT versus Δ*bapP* biofilm formation. Crystal violet assays were performed with varying concentrations (0, 1, 10 mM) of EDTA to measure biofilms at 24 h, 48 h, and 72 h. The significance level of selected t-test comparisons is shown above the bars, with * indicating $p < 0.001$.

A

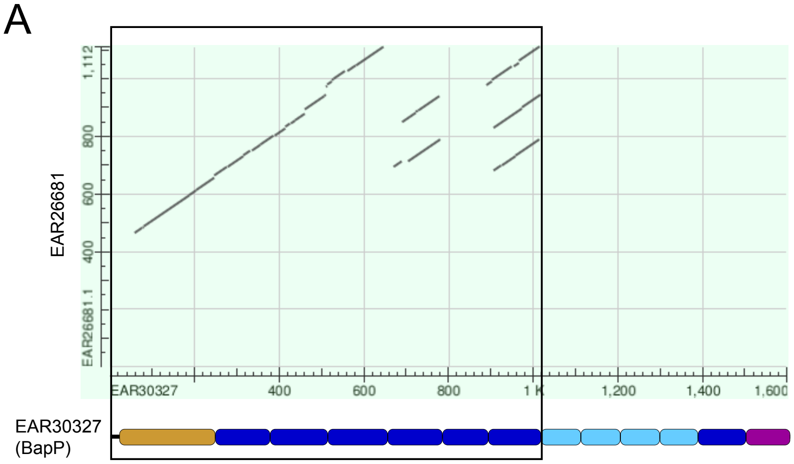

B

```
Score          Expect  Identities     Positives     Gaps
199 bits(506)  1e-55   190/664(29%)   307/664(46%)  95/664(14%)

Query  59    SDALAYQESTDRLYYVTKPVNG--KPRLVYVDMATEQHVDVAATTGTYRLAFSPDGQTLW  116
             SDA+AY +  DRLYY+TK VNG  KP++ YV+M T + V +A   G YRL FSPD   L+
Sbjct  466   SDAVAYDKINDRLYYITK-VNGALKPKVAYVNMQTGEDVKLAEIDGGYRLVFSPDSSQLF  524

Query  117   GSSEDTVFNINTNDGTVSNKVTLTGFATDADKLWGDIVFINDTLHIVTNKKLFAVDLAAG  176
               SS    +  IN    G V +K++L            GD+VFI D L+IV+   L  VDL
Sbjct  525   ASSGRDIVEINPTTGAVISKISLKSNDESITFGMGDLVFIQDELYIVSQLHLIKVDLVNK  584

Query  177   TVREVGMHNLS-VTGSTIDSLGQLLVSS-NAGNNKTDLYTLDPATAKPSLLSSINYRIND  234
                 +G H ++  TG+ +DS G LL+S  N    +T +Y+++     K + +++++Y IND
Sbjct  585   QATVLGKHGVNGATGAEVDSEGNLLISKINNATAQTTIYSINVTELKATAVATVDYAIND  644

Query  235   LATRTYVQPACT--------QLQDNVSSVEAIKNNSVEGQVLHARVHFEQPISTDTVTYL  286
             LA R +    C           ++ ++ ++E I +  VEG  L A+V F      +    +
Sbjct  645   LALRHFDGKTCNTDPVDPVEPIKSSLVAIELISDRQVEGDDLVAKVMFSG--GEEAQLNI  702

Query  287   NIENASAQKNADFDRFVELSFDNGMTWTS---VKNISTIATTDAFKGLSHFDVRIHSFKD  343
             N+ + +A  +ADF   V +SFD+G TW +     KN S      +A    L    ++IH+  D
Sbjct  703   NLSSNTADIDADFTSKVSVSFDDGQTWLTDIEAKNGSVTVPENAKSAL----IKIHTLTD  758

Query  344   GDIEGNENFVLE-AWNEGQADKKSRIFTIVDQDSSSPDVTSVTLTSENVPEGVFMVADVV  402
              ++E +E+E+  LE ++     +  KS + TIVD+ +                +P+   F+ A  +
Sbjct  759   TEVENDESLRLEVSFAHDPSVVKSELLTIVDKPTGGDTGGGDAC---EMPKVSFITALSI  815

Query  403   LSQATTSEFDHY------IQLVTNSENPAYSALNEDFTGQL-EISFNRGISWQSIGLVGE  455
              +    T +    +       +Q      + PA     N +F   +L +I    + + + ++   V
Sbjct  816   FNGEYTQDSKAFTYEGGEMQFEVGFDGPA--KCNGNFQFKLNDIETTQRLDYSALVSVSS  873

Query  456   LIKAR---------------IYEGVSEYKLRAKVYSDGVTEGAETAAISISASSDGLFAI  500
             L                   + EG    + +R K   +D    E   E    +S+    SD
Sbjct  874   LTNENWLNNVNVALGSATYAVQEGDEGFIVRVKTLADTTKEKNEVFTLSVWNKSDQSVDK  933

Query  501   ERPFTI-NDAV---------------------------KSC-----LPKVMYTIALP---  524
              +  +I N+A                              SC      +P + +  AL
Sbjct  934   YKDHSIENNAATDVDDGTPGTGGGTPGTGTPGNGNTDPDSCSSEDEIPAMKFITALSVDE  993

Query  525   -NPFS-EDGYMDFEVGYRSEAKCDGQYKFELVETSISYVDKAKKGVDFSTLVDIKDINSR  582
                F+ E G M++  G+  EA C G + F    +        D   KGVD+ST VDI+  + +
Sbjct  994   GRSFTKEGGKMEYYAGFAKEASCSGTFYFSFAD------DHTTKGVDYSTNVDIQTWD-K  1046

Query  583   EFEQFAVDASGVVTIDVPKGSAGFVVRVYGKPDDIIEGPEEFSINAWASPDQSDLFFKDI  642
             +  Q+ VDA+     ++V KG+AGF + +     DD  E  EE+ ++ W   D+SDL  KD
Sbjct  1047  QPAQYNVDAANGAAVNVVKGTAGFTITLTTLADDEAEEREEYFLHTWRKADKSDLKIKDH  1106

Query  643   TILD  646
             TI+D
Sbjct  1107  TIVD  1110
```

Figure S7. BLAST alignment dot plot depicting regions of similarity between BapP (x-axis) and a related *P. tunicata* protein (EAR26681). The region of similarity (boxed) covers the N-terminal domain and first six beta-sandwich repeats.

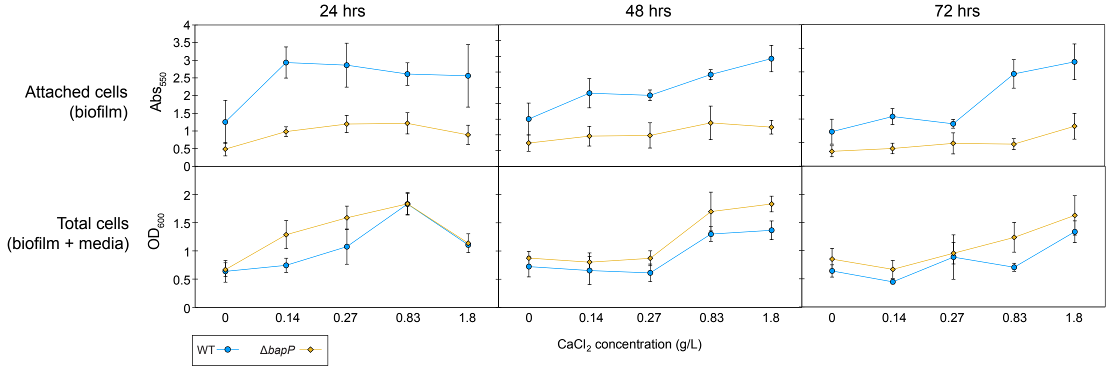

Figure S8. Line graphs depicting biofilm growth, quantified via 550 nm absorbance, and total cell growth, quantified via 600 nm absorbance, against added $CaCl_2$. Across all timepoints and $CaCl_2$ levels, the density of WT biofilms exceeds that of Δ*bapP* ($p < 0.05$, two-tailed t-test). However, the total cell growth (attached + non-attached cells) of Δ*bapP* matched or exceeded that of WT cultures. Error bars represent 95% confidence intervals.

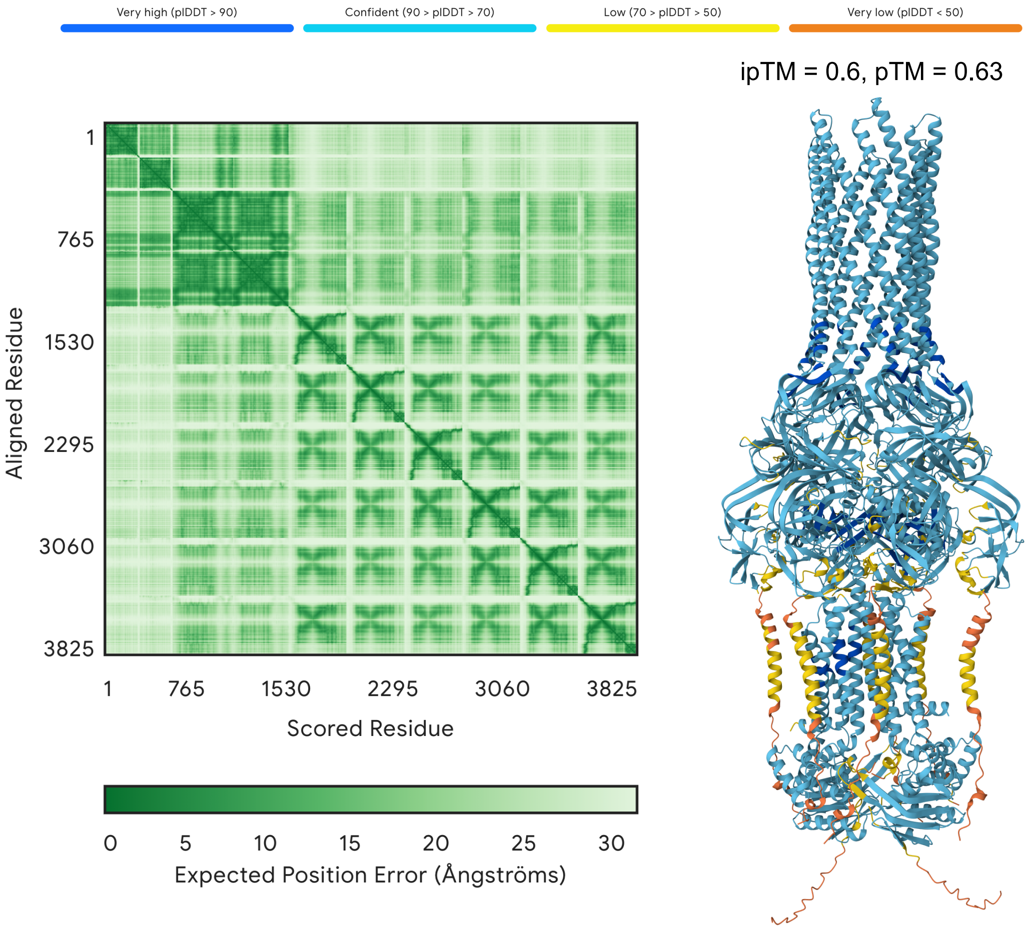

Figure S9. **Structural prediction of the EAR30320-EAR30323 complex produced by AlphaFold3.** Input sequences included six copies of EAR30323, two copies of EAR30322, and one copy each of EAR30321 and EAR30320. The AlphaFold3 heatmap depicting the pairwise interactions between all residues is shown on the left, with the structural model on the right colored by plDDT scores.

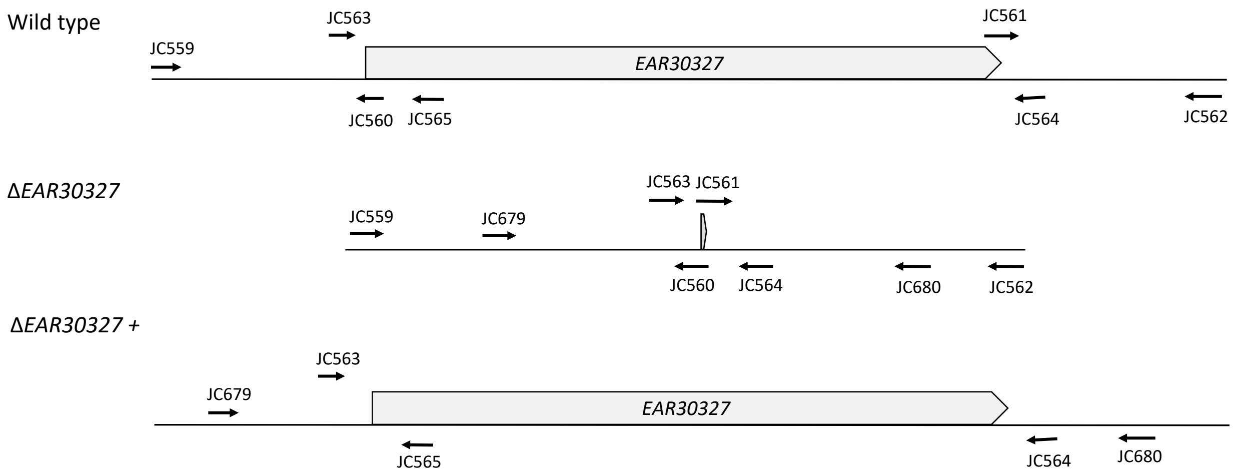

Figure S10. PCR primer binding regions in *P. tunicata* genomes. Top - the WT *P. tunicata* D2 genomic region containing the *bapP* gene (EAR30327). Middle – Δ*bapP* deletion mutant. Bottom – re-engineered strain in which the *bapP* gene was re-inserted into its original genomic location in the Δ*bapP* strain.

Supplementary Table Legends

Table S1. Shotgun proteomic (LC-MS/MS) protein abundance across all samples. All detected *P. tunicata* proteins are included.

Table S2. Top detected proteins with differential abundance in pellicle biofilms versus planktonic shaking samples.

Table S3. Top detected proteins with differential abundance in pellicle biofilms versus planktonic static samples.

Table S4. Top detected proteins with increased abundance in pellicle biofilms versus planktonic shaking and static samples.

Table S5. Top detected proteins with increased abundance in planktonic static samples.

Table S6. Bacterial strains and plasmids used in this work.

Table S7. Sequences of primers used in this work.
